# Candidate genes of SARS-CoV-2 gender susceptibility

**DOI:** 10.1038/s41598-021-01131-7

**Published:** 2021-11-09

**Authors:** Cristina Russo, Giovanna Morello, Roberta Malaguarnera, Salvatore Piro, Debora Lo Furno, Lucia Malaguarnera

**Affiliations:** 1 Department of Biomedical and Biotechnological Sciences, University of Catania, Catania, Italy; 2 Institute for Research and Biomedical Innovation (IRIB), Italian National Research Council (CNR), Catania, Italy; 3 Faculty of Medicine and Surgery, “Kore” University of Enna, Enna, Italy; 4 Department of Clinical and Molecular Medicine, University of Catania, Catania, Italy

**Keywords:** Computational biology and bioinformatics, Genetics, Immunology

## Abstract

The severe acute respiratory syndrome coronavirus (SARS-CoV-2) initiated a global viral pandemic since late 2019. Understanding that Coronavirus disease (COVID-19) disproportionately affects men than women results in great challenges. Although there is a growing body of published study on this topic, effective explanations underlying these sex differences and their effects on the infection outcome still remain uncertain. We applied a holistic bioinformatics method to investigate molecular variations of known SARS-CoV-2 interacting human proteins mainly expressed in gonadal tissues (testis and ovary), allowing for the identification of potential genetic targets for this infection. Functional enrichment and interaction network analyses were also performed to better investigate the biological differences between testicular and ovarian responses in the SARS-CoV-2 infection, paying particular attention to genes linked to immune-related pathways, reactions of host cells after intracellular infection, steroid hormone biosynthesis, receptor signaling, and the complement cascade, in order to evaluate their potential association with sexual difference in the likelihood of infection and severity of symptoms. The analysis revealed that within the testis network TMPRSS2, ADAM10, SERPING1, and CCR5 were present, while within the ovary network we found BST2, GATA1, ENPEP, TLR4, TLR7, IRF1, and IRF2. Our findings could provide potential targets for forthcoming experimental investigation related to SARS-CoV-2 treatment.

## Introduction

In early December 2019, a new transmissible infection spread widely in China^1^, which caused a respiratory illness termed severe acute respiratory syndrome coronavirus 2 (SARS-CoV-2)^2^. On March 11th, 2020 the World Health Organization (WHO) declared SARS-CoV-2 a global viral pandemic disease^3^. The SARS-CoV-2 infection is very heterogeneous disease that has affected millions of people worldwide. It is characterized by a broad clinical spectrum, encompassing asymptomatic infection, mild upper respiratory tract infection, acute respiratory distress syndrome (ARDS), septic shock, multi-organ failure, and death^4^. Since COVID-19 is a massive population pathology, the SARS-CoV-2 can be detected in the wastewater^5^. Therefore, significant number of patients could have digestive tract infection leading to the urinary and genital infections^6^. Nevertheless, the viremia at the urine infection is detectable in about 10% of the patients. Hence, the majority of the cases the infection is confined to the respiratory tract SARS-CoV-2 viral load results in a worsen prognosis^7^. COVID-19 virus attacks typically the mucosal membranes of the host through the N-terminal peptidase domain of angiotensin-converting enzyme 2 (ACE2) at the surface of the cell membrane using the S domain^8^. ACE2 protein is expressed in systemic tissues including lung, heart, stomach, kidney, ileum, colon, thyroid, genitourinary and adipose tissue^9^. Consequently, any cells expressing ACE2 is likely to SARS-CoV-2 infection. Since ACE2 is also expressed in spermatogonia, Leydig and Sertoli cells, testicular tissue is a target tissue of SARS-CoV-2^10^. After the binding of SARS-CoV-2 to ACE2 and virion membrane fusion, downregulation of ACE2 expression generates an excessive synthesis of angiotensin by the related enzyme^11^. An augmented replication of coronaviruses downregulates the expression of ACE2^12^. AT2 cells are the principal target cells of SARS-CoV-2. However, the lung expresses moderate levels of ACE2. Additionally, SARS-CoV-2 requires co-receptor or supplementary membrane proteins such as cellular protease transmembrane protease serine-2 (TMPRSS2) to facilitate its infection^13^. Moreover, Alanyl (membrane) Aminopeptidase (ANPEP), Glutamyl Aminopeptidase (ENPEP) and Dipeptidyl peptidase-4 (DPP4) are additional genes correlated with ACE2^14^. ANPEP is mainly expressed in the colon, ileum, rectum, kidney, liver and skin. ENPEP, a member of the peptidase M1 family, is the mammalian type II integral membrane zinc-containing endopeptidases. ENPEP controls blood pressure regulation and blood vessel development through of the renin-angiotensin system pathway^15^. The connection between ENPEP and viral infection is still mysterious. Severe acute respiratory syndrome coronavirus 2 pandemic capacity is derived from the unique structural features on its spike protein: fast viral surfing over the epithelium with flat N‐terminal domain, tight binding to ACE2 entry receptor, and furin protease utilization. In addition, the possible involvement of other components such as lipid rafts, CLRs, and neuropilin is, in combination, mediating the accelerated cell entry and other critical steps in its overwhelming contagious capacity and pandemy. Recently it has been proposed that SARS‐CoV‐2 can move rapidly over the cell surface of the lung epithelium interacting with glycalyx sialic acid through its S protein N terminal domain^16^. The pandemic capacity of SARS Cov 2 is also caused by the binding to the lectin receptors of type C (CLR) exploiting the viral escape‐based “detour” entry without cleavage by protease and neuropilin 1 (NRP1) avoiding the involvement of ACE2. In addition other proteases S protein interacting through its polybasic S1/S2 with surface sugars and receptors and may in theory be using three or more different pathways for cell entry^16^. Therefore, these unique interactions of the SARS‐CoV‐2 are believed to be the critical pandemic capacity factors. All these genes encode peptidases, which, for mysterious reasons, are emploied by coronavirus as their receptors. Host immune response is essential in the battle against viruses. Commonly, the infected cells produce interferons to destroy viral activities^17^. In addition, interferons stimulate neighboring cells to upregulate MHC class I molecules making CD8 + T cells able to detect and remove the viral infection^18^. To date, two questions remain unresolved, namely low prepuberal mortality rates^19^ and that COVID-19 disproportionally affects men and women^20^. Men with COVID-19 show an increased risk of complications compared to women (~ 58% vs 42%)^4^. It has been reported that male have potential risks infertility due to the mild infections at the testicles^21^. Moreover, meta-analysis studies reported that an increased proportion of men affected by COVID-19 required hospitalization compared with women^22^. Therefore, male patients contracting the SarsCov2 virus die at twice the rate of females (Table 1)^7,22^. But only half of them shows viremia which is present in 13% of the symptomatic but non-hospitalized people^7^. Despite sexual differences in severity and mortality have been associated with a greater incidence of comorbidities (i.e. chronic lung disease, cardiovascular disease, hypertension and diabetes) and high-risk behaviors comprising smoking and alcohol use in men^23^. These disproportions persisted even after checking for these potential confounders. Sex differences in the immune response are well-known. Indeed, men and women react in a different way to foreign and self-antigens^24^. Hospitalized patients with SARS-CoV-2 infection have shown higher levels of chemokines, C-reactive protein (CRP) stimulating “cytokine storm”,characterised by significant production of IL-6, from the ongoing infection in the lungs, with sex differences existing in immune responses^7,25^. The genital infections is not be the case in all SARS-CoV-2 fatalities^7^. To date, a number of hypotheses have been proposed to clarify the male susceptibility to severe COVID-19 infection, nevertheless, the basic mechanisms or factors of the noticed sex differences have not been fully explained. Some theories have been postulated, one is the effect of sexual hormones affecting immune responses, therefore estrogen reinforces the immune system and testosterone weaken it^13^, another is the action of androgen on target tissues, such as the lung^26^. Moreover, sex differences may occur in ACE 2 receptor and the cellular serine protease TMPRSS2, which are responsible for viral entrance and priming, respectively^13^. Knowledge of gender differences in terms of disease severity, clinical characteristics and mortality is a key point for better disease management, the unfavourable course of the disease prediction and the intervention strategies for both men and women. In this study, we explored the gender differences in the transcriptional landscape of the host response to SARS-CoV-2. In particular, we investigated the expression of known SARS-CoV-2 interacting human proteins and their interaction in the testicles and ovarian tissues, to provide an immediate understanding of variable incidence to SARS-CoV-2 infection and severity of symptoms in men and women.

**Table 1:** Differences of SARS-CoV-2 gender susceptibility in different countries (https://globalhealth5050.org/the-sex-gender-and-covid-19-project/).

Country	Country	Country	Country	Cases (male)	Cases (female)	Deaths date	Deaths were sex-disaggregated	Deaths (male)	Deaths (female)	Deaths in confirmed cases date	Proportion of deaths in confirmed cases (male)	Proportion of deaths in confirmed cases (female)	Proportion of deaths in confirmed cases (Male:female ratio)
Afghanistan	Yes	21/09/2021	122,400	59%	41%	21/09/2021	3888	65%	35%	21/09/2021	4%	3%	1,31
Albania	Yes	10/09/2021	164,276	48%	52%	10/09/2021	2594	67%	33%	10/09/2021	2%	1%	2,2
Algeria	Partial	20/05/2021	126,156	54%	46%								
Argentina	Yes	22/09/2021	5,202,330	49%	51%	21/09/2021	112,361	58%	42%	21/09/2021	3%	2%	1,42
Australia	Yes	22/09/2021	91,832	52%	48%	22/09/2021	1200	51%	49%	22/09/2021	1%	1%	0,96
Austria	Yes	23/09/2021	726,873	49%	51%	23/09/2021	10,716	53%	47%	23/09/2021	2%	1%	1,18
Azerbaijan	Partial	06/09/2021	445,278	49%	51%								
Bahrain	Partial	06/07/2020	6081	88%	12%								
Bangladesh	Yes	21/09/2021	1,544,238	71%	29%	21/09/2021	27,251	77%	23%	21/09/2021	2%	1%	1,37
Barbados	Yes	23/09/2021	7065	50%	50%	02/02/2021	13	62%	38%	02/02/2021	1%	1%	1,12
Belgium	Yes	20/09/2021	1,223,766	47%	53%	21/09/2021	25,499	51%	49%	21/09/2021	2%	2%	1,17
Belize	Yes	20/09/2021	18,902	51%	49%	20/09/2021	395	64%	36%	20/09/2021	3%	2%	1,7
Bermuda	Yes	12/08/2021	2663	45%	55%	22/09/2021	45	56%	44%	12/08/2021	1%	1%	1,38
Bhutan	Yes	20/09/2021	2597	61%	39%	20/09/2021	3	67%	33%	20/09/2021			1,26
Bosnia and Herzegovina	Yes	21/09/2021	148,518	52%	48%	21/09/2021	5730	61%	39%	21/09/2021	5%	3%	1,46
Botswana	Partial	17/07/2020	48	83%	17%	28/09/2020	15	40%	60%				
Brazil	Yes	19/12/2020	565,465	56%	44%	11/09/2021	534,119	56%	44%	19/12/2020	33%	31%	1,06
Bulgaria	Partial	25/05/2020	2443	49%	51%								
Burkina Faso	Yes	20/09/2021	14,052	63%	37%	25/08/2020	55	75%	25%	25/08/2020	5%	3%	1,54
Cabo Verde	Yes	20/09/2021	36,924	47%	53%	11/07/2021	286	48%	52%	27/12/2020	1%	1%	1,53
Cambodia	Yes	03/05/2021	15,351	43%	57%	03/05/2021	106	57%	43%	03/05/2021	1%	1%	1,76
Canada	Yes	16/09/2021	1,552,308	50%	50%	16/09/2021	27,180	51%	49%	16/09/2021	2%	2%	1,03
Cayman Islands	Partial	16/12/2020	302	53%	47%								
Central African Republic	Partial	16/05/2021	7010	68%	32%								
Chad	Yes	17/07/2021	3697	69%	31%	16/05/2021	114	79%	21%	16/05/2021	4%	3%	1,4
Chile	Yes	22/09/2021	1,964,150	50%	50%	07/05/2020	294	60%	40%	07/05/2020	1%	1%	1,32
China	Yes	28/02/2020	55,924	51%	49%	28/02/2020	2114	64%	36%	28/02/2020	5%	3%	1,68
Colombia	Yes	20/09/2021	4,942,249	48%	52%	20/09/2021	125,924	61%	39%	20/09/2021	3%	2%	1,73
Congo	Partial	15/11/2020	5632	72%	28%								
Costa Rica	Yes	21/09/2021	521,182	50%	50%	21/09/2021	6098	61%	39%	21/09/2021	1%	1%	1,56
Croatia	Partial	19/09/2021	392,248	47%	53%								
Cuba	Partial	13/12/2020	9492	53%	47%	17/07/2021	1906	57%	43%				
Cyprus	Partial	21/09/2021	117,926	50%	50%								
Czech Republic	Yes	21/09/2021	1,665,737	49%	51%	15/09/2021	30,417	57%	43%	19/07/2021	2%	2%	1,43
Denmark	Yes	21/09/2021	355,603	50%	50%	21/09/2021	2633	54%	46%	21/09/2021	1%	1%	1,19
Djibouti	Partial	02/06/2020	3779	68%	32%								
Dominican Republic	Yes	28/08/2020	93,732	51%	49%	30/08/2020	1710	66%	34%	28/08/2020	2%	1%	1,88
Ecuador	Yes	25/07/2021	474,212	51%	49%	25/07/2021	30,569	53%	47%	25/07/2021	7%	6%	1,07
El Salvador	Partial	22/09/2021	102,024	50%	50%								
England	Yes	23/09/2021	6,646,405	48%	52%	23/09/2021	158,152	56%	44%	23/09/2021	3%	2%	1,39
Equatorial Guinea	Yes	15/09/2021	11,063	58%	42%	07/06/2021	113	67%	33%	07/06/2021	2%	1%	1,47
Estonia	Yes	20/09/2021	149,097	46%	54%	13/09/2021	1313	52%	48%	14/06/2021	1%	1%	1,24
Eswatini	Yes	18/04/2021	18,417	48%	52%	20/04/2021	671	52%	48%	20/04/2021	4%	3%	1,14
Ethiopia	Partial	29/06/2020	5846	61%	39%								
Faroe Islands	Partial	21/09/2021	944	54%	46%								
Finland	Yes	21/09/2021	137,117	53%	47%	21/09/2021	1059	54%	46%	21/09/2021	1%	1%	1,04
France	Yes	18/09/2021	6,737,561	47%	53%	16/09/2021	88,518	58%	42%	16/09/2021	2%	1%	1,55
French Polynesia	Partial	04/10/2020	2228	49%	51%	29/09/2020	7	43%	57%				
Gabon	Partial	26/08/2020	8409	40%	60%								
Gambia	Partial	19/09/2021	9775	59%	41%								
Germany	Yes	20/09/2021	4,126,341	49%	51%	20/09/2021	92,919	53%	47%	20/09/2021	2%	2%	1,17
Ghana	Partial	21/09/2021	124,309	58%	42%								
Gibraltar	Partial					06/01/2021	10	80%	20%				
Greece	Yes	22/09/2021	634,298	51%	49%	22/09/2021	14,575	57%	43%	22/09/2021	3%	2%	1,27
Guatemala	Yes	20/09/2021	531,650	51%	49%	20/09/2021	13,115	66%	34%	20/09/2021	3%	2%	1,88
Guernsey	Partial	11/05/2020	252	37%	63%	03/02/2021	13	38%	62%				
Guinea	Partial	23/03/2021	20,267	66%	34%								
Guinea-Bissau	Yes	20/06/2021	3825	60%	40%	20/06/2021	69	70%	30%	20/06/2021	2%	1%	1,5
Guyana	Partial	20/09/2021	29,647	48%	52%								
Haiti	Yes	15/09/2021	116,433	54%	46%	12/06/2021	361	57%	43%	12/06/2021	2%	2%	1,1
Honduras	Partial	14/09/2020	67,789	52%	48%								
Hong Kong	Yes	20/09/2021	12,166	48%	52%	20/09/2021	213	59%	41%	20/09/2021	2%	1%	1,57
Iceland	Partial	21/06/2020	1823	50%	50%								
India	Yes	18/05/2021	24,766,088	61%	39%	21/05/2020	3711	64%	36%	06/04/2020	3%	3%	0,85
Indonesia	Yes	21/09/2021	4,195,958	49%	51%	21/09/2021	140,805	52%	48%	21/09/2021	4%	3%	1,16
Iran	Yes	17/03/2020	14,991	57%	43%	17/03/2020	853	59%	41%	17/03/2020	6%	5%	1,09
Iraq	Yes	20/09/2021	1,978,412	61%	39%	20/09/2021	21,869	58%	42%	20/09/2021	1%	1%	0,87
Isle of Man	Partial	20/09/2021	7208	51%	49%								
Israel	Yes	20/09/2021	1,219,265	48%	52%	20/09/2021	7555	56%	44%	20/09/2021	1%	1%	1,42
Italy	Yes	15/09/2021	4,616,789	49%	51%	15/09/2021	129,558	56%	44%	15/09/2021	3%	2%	1,35
Jamaica	Yes	21/09/2021	81,828	43%	57%	20/09/2021	1772	52%	48%	20/09/2021	3%	2%	1,42
Japan	Partial	22/06/2020	17,301	55%	45%	21/09/2021	13,522	58%	42%				
Jersey	Yes	23/09/2021	9885	51%	49%	23/09/2021	78	62%	38%	23/09/2021	1%	1%	1,56
Jordan	Yes	22/09/2021	764,998	50%	50%	22/09/2021	10,637	60%	40%	22/09/2021	2%	1%	1,52
Kazakhstan	Partial	08/06/2020	9452	61%	39%								
Kenya	Yes	19/09/2021	245,680	58%	42%	19/09/2021	4993	65%	35%	19/09/2021	2%	2%	1,32
Kosovo	Yes	21/09/2021	159,488	47%	53%	21/09/2021	2917	59%	41%	21/09/2021	2%	1%	1,62
Kyrgyzstan	Yes	16/07/2020	12,498	47%	53%	16/07/2020	155	62%	38%	16/07/2020	2%	1%	1,83
Latvia	Yes	22/09/2021	151,917	44%	56%	22/09/2021	2665	50%	50%	22/09/2021	2%	2%	1,29
Lebanon	Partial	15/02/2021	339,122	54%	46%	06/07/2020	36	31%	69%				
Liberia	Yes	17/07/2021	5396	65%	35%	17/07/2021	148	66%	34%	17/07/2021	3%	3%	1,04
Liechtenstein	Partial	20/09/2021	3301	49%	51%								
Lithuania	Yes	21/09/2021	314,926	44%	56%	21/09/2021	4830	49%	51%	21/09/2021	2%	1%	1,23
Luxembourg	Yes	22/09/2021	77,552	50%	50%	22/09/2021	835	55%	45%	22/09/2021	1%	1%	1,22
Malawi	Yes	07/02/2021	27,422	59%	41%	05/01/2021	196	76%	24%	05/01/2021	3%	2%	1,5
Maldives	Yes	21/09/2021	83,311	59%	41%	21/09/2021	229	60%	40%	21/09/2021			1,05
Mexico	Yes	22/09/2021	3,596,279	50%	50%	22/09/2021	273,377	62%	38%	22/09/2021	9%	6%	1,63
Moldova	Yes	19/09/2021	282,650	41%	59%	19/09/2021	6605	49%	51%	19/09/2021	3%	2%	1,35
Montenegro	Partial	21/09/2021	126,837	49%	51%	15/03/2021	83,685	49%	51%				
Morocco	Yes	18/07/2020	17,015	53%	47%	21/09/2020	1855	66%	34%	18/07/2020	3%	2%	1,8
Myanmar	Yes	10/09/2020	2265	53%	47%	28/09/2020	226	64%	36%	01/09/2020	1%		3,84
Nepal	Yes	21/09/2021	782,180	59%	41%	06/09/2021	10,833	66%	34%	06/09/2021	2%	1%	1,32
Netherlands	Yes	20/09/2021	1,987,815	48%	52%	20/09/2021	18,128	55%	45%	20/09/2021	1%	1%	1,31
New Zealand	Yes	22/09/2021	4114	51%	49%	22/09/2021	27	52%	48%	22/09/2021	1%	1%	1,05
Nigeria	Yes	08/08/2021	160,006	60%	40%	08/08/2021	1569	71%	29%	08/08/2021	1%	1%	1,62
North Macedonia	Yes	22/09/2021	187,618	50%	50%	22/09/2021	6509	62%	38%	22/09/2021	4%	3%	1,63
Northern Ireland	Yes	22/09/2021	230,382	48%	52%	23/09/2021	2524	54%	46%	23/09/2021	1%	1%	1,28
Norway	Yes	22/09/2021	183,808	53%	47%	22/09/2021	850	54%	46%	22/09/2021			1,05
Pakistan	Yes	18/08/2020	289,832	74%	26%	18/08/2020	6190	74%	26%	18/08/2020	2%	2%	1,01
Panama	Yes	14/08/2020	79,402	54%	46%	05/06/2020	373	68%	32%	05/06/2020	3%	2%	1,47
Paraguay	Yes	14/03/2021	180,014	48%	52%	14/03/2021	3476	61%	39%	14/03/2021	2%	1%	1,66
Peru	Yes	20/09/2021	2,168,430	51%	49%	20/09/2021	199,226	64%	36%	20/09/2021	11%	7%	1,67
Philippines	Yes	21/09/2021	2,401,916	51%	49%	21/09/2021	37,074	56%	44%	21/09/2021	2%	1%	1,26
Poland	Partial					05/02/2021	11,451	57%	43%				
Portugal	Yes	22/09/2021	1,063,252	46%	54%	22/09/2021	17,933	52%	48%	22/09/2021	2%	1%	1,28
Republic of Ireland	Yes	18/09/2021	375,259	49%	51%	18/09/2021	5193	53%	47%	18/09/2021	1%	1%	1,17
Romania	Yes	19/09/2021	1,152,052	46%	54%	19/09/2021	35,592	57%	43%	19/09/2021	4%	2%	1,57
Rwanda	Yes	21/09/2021	95,503	49%	51%	21/09/2021	1215	55%	45%	21/09/2021	1%	1%	1,28
Saint Lucia	Yes	17/09/2021	10,237	45%	55%	08/03/2021	39	87%	13%	31/01/2021	2%		6,73
Scotland	Yes	19/09/2021	535,527	48%	52%	19/09/2021	10,826	51%	49%	19/09/2021	2%	2%	1,13
Serbia	Partial					03/05/2020	193	62%	38%				
Sierra Leone	Partial	21/09/2021	6393	59%	41%								
Singapore	Partial	05/05/2020	6530	89%	11%								
Slovakia	Yes	20/09/2021	403,802	48%	52%	16/07/2021	12,455	54%	46%	15/06/2021	4%	3%	1,27
Slovenia	Yes	22/09/2021	287,176	47%	53%	19/09/2021	4824	49%	51%	15/08/2021	2%	2%	1,07
Somalia	Partial	22/09/2021	19,235	71%	29%								
South Africa	Yes	22/09/2021	2,857,730	43%	57%	22/09/2021	91,959	48%	52%	22/09/2021	4%	3%	1,23
South Korea	Yes	21/09/2021	289,263	52%	48%	21/09/2021	2413	50%	50%	21/09/2021	1%	1%	0,92
Spain	Yes	22/09/2021	4,932,891	48%	52%	22/09/2021	85,832	55%	45%	22/09/2021	2%	2%	1,33
Sweden	Yes	22/09/2021	1,147,876	49%	51%	23/09/2021	14,813	55%	45%	23/09/2021	1%	1%	1,25
Switzerland	Yes	22/09/2021	830,013	48%	52%	22/09/2021	10,643	54%	46%	22/09/2021	1%	1%	1,26
Taiwan	Yes	21/09/2021	16,152	51%	49%	20/04/2021	11	82%	18%	20/04/2021	2%		4,27
Thailand	Yes	01/11/2020	3784	56%	44%	01/04/2021	93	75%	25%	01/11/2020	2%	1%	2,49
Tunisia	Yes	10/01/2021	162,350	44%	56%	10/01/2021	5310	66%	34%	10/01/2021	5%	2%	2,47
Turkey	Yes	25/10/2020	362,800	51%	49%	25/10/2020	9799	62%	38%	25/10/2020	3%	2%	1,56
USA	Yes	20/09/2021	32,683,710	48%	52%	15/09/2021	658,754	55%	45%	09/09/2021	2%	2%	0,77
Uganda	Yes	09/07/2021	48,646	61%	39%	09/07/2021	364	69%	31%	09/07/2021	1%	1%	1,43
Ukraine	Yes	24/09/2021	2,379,483	40%	60%	22/09/2021	55,424	53%	47%	22/09/2021	3%	2%	1,69
Venezuela	Partial	22/09/2021	358,462	51%	49%								
Vietnam	Yes	12/09/2021	475,831	48%	52%	11/07/2021	86	43%	57%	11/07/2021			0,96
Wales	Yes	22/09/2021	335,046	46%	54%	22/09/2021	5837	56%	44%	22/09/2021	2%	1%	1,48
Yemen	Yes	20/05/2021	6617	64%	36%	20/05/2021	1256	70%	30%	20/05/2021	21%	16%	1,31
Zimbabwe	Yes	12/12/2020	11,219	55%	45%	12/12/2020	307	62%	38%	12/12/2020	3%	2%	1,33

## Materials and methods

### Ethical compliance

This investigation does not contain any studies with human participants or animals performed by any of the authors, and therefore no ethical compliance is required.

### Data selection by SARS-CoV-2 Human protein atlas (HPA) program

The SARS-CoV-2 HPA program (HPA, https://www.proteinatlas.org/humanproteome/sars-cov-2) provides a freely available information source on the tissue and cellular expression patterns of known SARS-CoV-2 interacting human proteins, based on transcriptomics and antibody-based proteomics. Genes associated with inflammatory processes, such as chemokines, cytokines, metalloproteinase, as well as bone marrow stromal antigen 2 or tetherin (BST2), ANPEP, ENPEP, forkhead box P3 (FOXP3), GATA Binding Protein 1 (GATA1), High Mobility Group1 (HMGB1), Interferon Regulatory Factor (IRF1 and 2), and Serine/Cysteine Proteinase Inhibitor Clade G Member 1 (SERPING1), were integrated with the list of SARS-CoV-2 HPA program filtered on the basis of their selective expression on testis and ovary tissue. Moreover, the androgen and estrogen receptors (AR and ER) were also considered.

### In silico gene–gene interaction using GeneMANIA

The total list of genes belonging to the testicles or ovaries were given to the GeneMANIA plugin in Cytoscape to evaluate their interactome and possible biological functions. GeneMANIA is a Gene Multiple Association Network Integration Algorithm and tests the weights from data sources based on their predicted value to re-establish the query list. GeneMANIA generates speculation regarding gene function, analyzing gene lists, and prioritizing functional genes for functional evaluation. It then expands the query list with practically identical genes that have common characteristics with the initial query genes and display an interactive, applicable association network, allowing the genes to reveal the relationship between datasets^27^. Cytoscape (version:3.8.2, http://www.cytoscape.org/) was used to visualize and analyze the protein–protein interaction (PPI) network. This software allows users to construct a composite gene–gene functional interaction network from input lists of genes, taking into account experimental and in silico interactions^28^. In particular, the GeneMANIA app uses association data, including protein and genetic interactions, pathways, co-expression and co-localization similarity information and protein domain similarity data. In a given network, each gene is represented as a node and the interactions between the nodes are defined as edges. Topological parameters of the networks were analyzed using the Network-Analyzer plugin in Cytoscape. In particular, in this study, we focused on the degree of centrality of a node, representing the number of edges linked to a given node. In this context, nodes having a high degree of centrality represent the network hub genes. Finally, we focused our analysis on the direct interaction between SARS-CoV-2 interacting human proteins, other immune/inflammation-associated candidates and the androgen and estrogen receptors on testis- and ovary-associated networks, respectively.

### Functional enrichment analysis

To identify biological process terms that are overrepresented in the list of proteins in both testis- and ovary-associated networks, a Gene Ontology (GO) functional enrichment analysis was performed using the Biological Networks Gene Ontology tool (BiNGO, version 3.0.3) in Cytoscape v.3.8.2 (http://www.cytoscape.org/)^29^. Significantly enriched GO terms were identified using hypergeometric tests and corrected by Benjamini and Hochberg false discovery rate (FDR) adjustment, and p ≤ 0.05 was applied as a cutoff for statistical significance.

## Results

### Sex is a discriminating index in human proteins interacting in testicles and ovaries of SARS-CoV-2 patients

**Figure 1:**
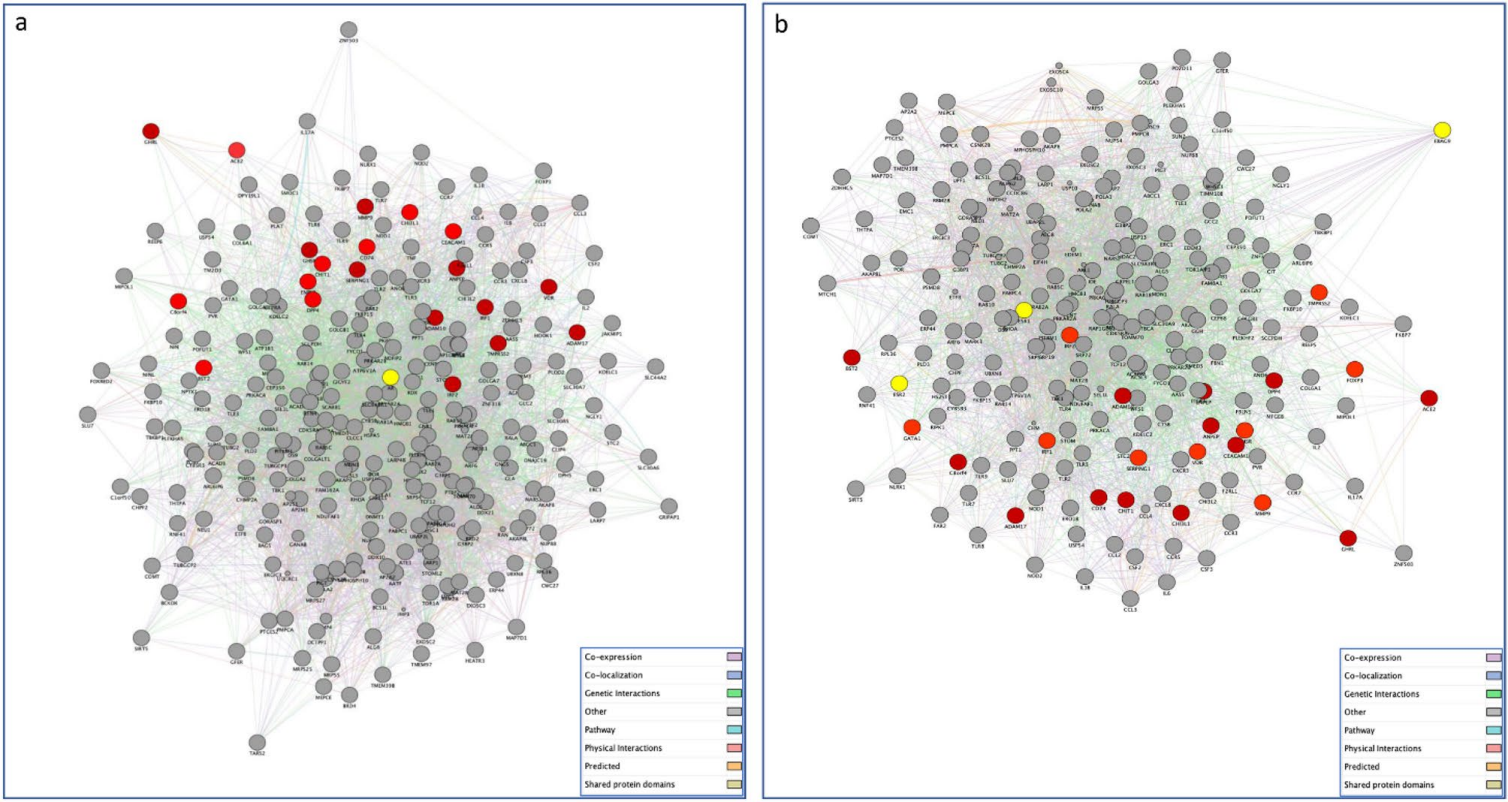
Gene networks of SARS-CoV-2 targeted human proteins expressed in testis (**a**) and ovary (**b**) and their interactions with the list of genes of our interest. The PPI network constructed by GeneMANIA shows the relationships for SARS-CoV-2 targeted human proteins expressed in testis (**a**) and ovary (**b**) and the genes of interest (nodes) connected (with edges) according to the functional association networks from the databases. Differently colored ‘edges’ indicate the type of evidence supporting each interaction: co-expression (light purple), physical interaction (pink), genetic interaction (green), shared protein domains (golden yellow), pathway (light blue), predicted (orange), and co-localization (blue).

**Figure 2:**
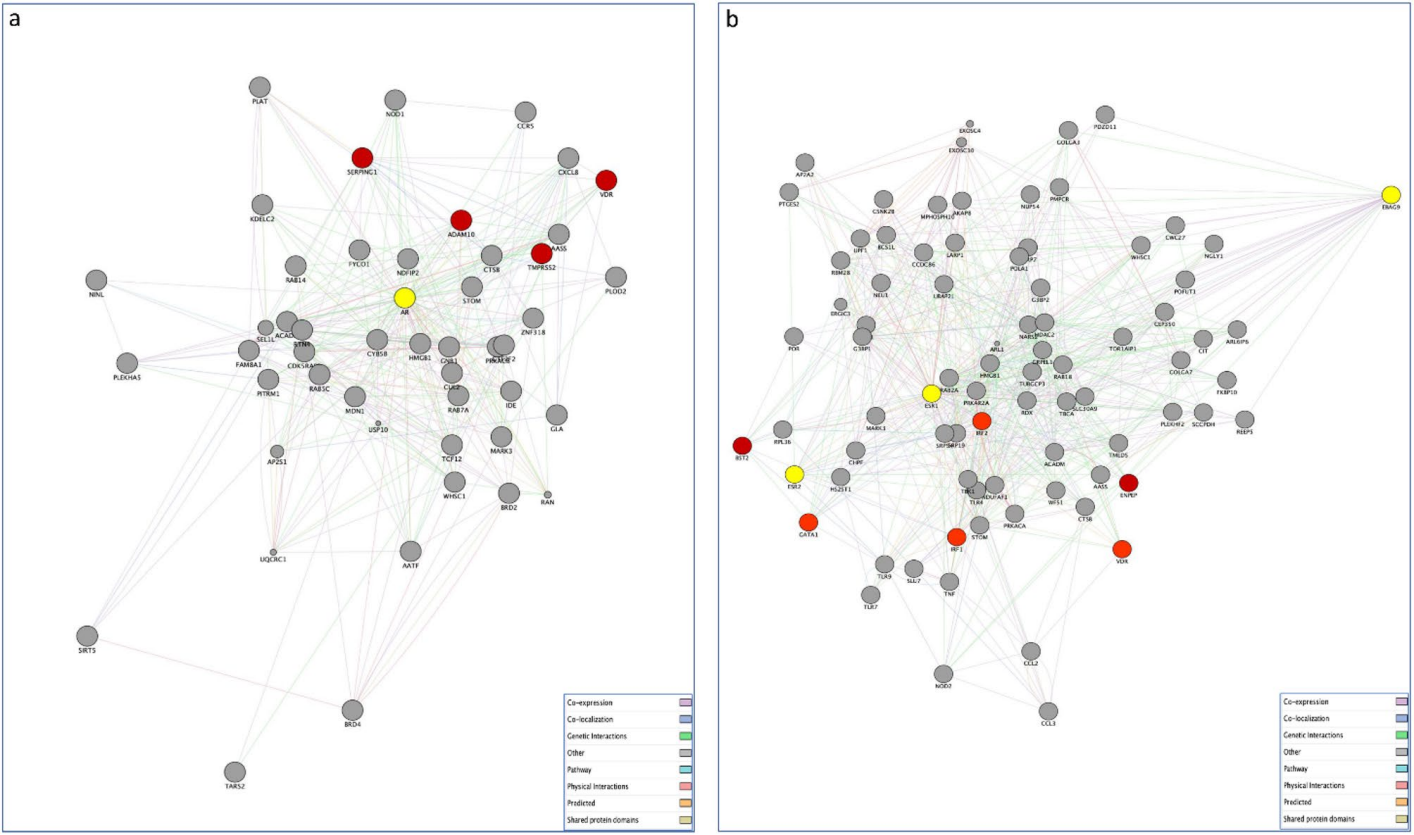
Gene networks of SARS-CoV-2 targeted human proteins expressed in testis (**a**) and ovary (**b**) and their interactions with the list of genes of our interest and gonadal steroid hormones. The PPI network constructed by GeneMANIA shows the interconnection between SARS-CoV-2 targeted human proteins expressed in testis (**a**) and ovary (**b**), the genes of interest and androgen and estrogen receptors, respectively. The edges between nodes (proteins) indicate interactions based on the GeneMANIA database information. Differently colored ‘edges’ indicate the type of evidence supporting each interaction: co-expression (light purple), physical interaction (pink), genetic interaction (green), shared protein domains (golden yellow), pathway (light blue), predicted (orange), and co-localization (blue). A detailed view of both (**a**) and (**b**) networks for each different interaction type is provided in Supplementary Fig. S1.

As expected, our analysis highlighted a difference in the number of the SARS-CoV-2 interacting human proteins selectively expressed in gonadal tissue, with a total of 386 protein-encoding genes for testicles and 268 genes for ovaries. This preliminary data show that there are more human proteins interacting with SARS-CoV-2 in the testicles than in the ovaries. We then used the Cytoscape GeneMANIA application to construct gene networks of the proposed SARS-CoV-2 targeted human proteins expressed in both the testis and ovary to investigate their interaction with our genes of interest (Fig. 1a,b) and with the androgen and estrogen receptors, respectively (Fig. 2a,b). The testis-associated PPI network consisted of 267 nodes (proteins) and 5245 edges (interactions). In particular, the majority of connections were co-expression interactions (55.26%), followed by physical interactions (21.45%), 9.67% of predicted protein interactions, 6.28% with co-localisation, 4% with genetic interactions, 2.11% pathway and 1.24% with shared protein domains. Furthermore, the ovary-related network included 223 nodes and 3726 edges. Also in this case, the majority of connections were co-expression interactions (47.79%), followed by physical interactions (30.83%), 8.36% of predicted protein interactions, 6.91% with co-localisation, 3.13% with genetic interactions, 1.57% pathway and 1.41% with shared protein domains. Next, for clarity, we filtered all the testis- and ovary-associated networks focusing our attention on the direct interactions with hormone receptor genes, AR and Estrogen Receptor Binding Site Associated Antigen 9 (EBAG9), ESR1, ESR2, respectively. The resulting networks consisted of 49 nodes (318 edges) for the testis-related network and 82 nodes (634 edges) for the ovary-related network.

### Functional enrichment analysis and identification of key genes

To obtain a more in-depth understanding of the biological processes associated with all the PPI networks, GO enrichment analysis was performed using the BINGO plugin in Cytoscape. The over-represented GO terms (adjusted P < 0.05) were mainly associated with the regulation of cell activation, leukocyte activation, peptide transport, and immune response for both ovary and testis-related network (Tables 2 and 3).

**Table 2:** GO enrichment analysis Ovary Network.

GO I.D	Background genes	Genes	Description	FDR value	P-value
GO.0060135	60	6	Maternal process involved in female pregnancy	9.2E−4	9.9E−5
GO.0046631	60	6	Alpha–beta T cell activation	9.2E−4	9.9E−5
GO.0009755	171	8	Hormone-mediated signaling pathway	0.0064	9.9E−4
GO.0002757	332	15	Immune response-activating signal transduction	1.3E−4	9.93E−6
GO.0043434	362	14	Response to peptide hormone	9.2E−4	9.91E−5
GO.0051259	512	15	Protein complex oligomerization	0.0064	9.8E−4
GO.0030155	623	17	Regulation of cell adhesion	0.0064	9.8E−4
GO.0002705	109	10	Positive regulation of leukocyte mediated immunity	1.95E−5	9.84E−7
GO.0001819	390	25	Positive regulation of cytokine production	1.03E−9	9.7E−12
GO.0031347	676	31	Regulation of defense response	7.22E−9	9.6E−11

**Table 3:** GO enrichment analysis testis network.

GO I.D	Background genes	Genes	Description	FDR value	P-value
GO.0044257	562	23	Cellular protein catabolic process	9.93E−5	4.75E−6
GO.0051050	892	33	Positive transport regulation	9.78E−6	3.36E−7
GO.0002730	4	3	Regulation of dendritic cell cytokine production	9.6E−4	8.11E−5
GO.0007052	70	7	Mitotic spindle organization	9.5E−4	7.98E−5
GO.0002702	84	9	Positive regulation of production of molecular mediator of immune response	9.56E−5	4.55E−6
GO.0036230	502	33	Granulocyte activation	9.47E−11	3.14E−13
GO.0002682	1391	49	Regulation of immune system process	9.46E−8	1.45E−9
GO.0006508	1203	37	Proteolysis	9.46E−5	4.49E−6
GO.0051704	2514	77	Multi-organism process	9.42E−10	6.24E−12
GO.0032722	56	11	Positive regulation of chemokine production	9.35E−8	1.41E−9

The subsequent network topological analysis identified 4 hub genes common to both networks, highlighted in bold form into Table 4, with radixin (RDX) showing the higher connectivity degrees in both PPI networks (n = 92 in the testis network and n = 80 in the ovary network), followed by HMGB1 (n = 87 in the testis network and n = 72 in the ovary network), RAB5C (n = 81 in the testis network and n = 69 in the ovary network) and IRF2 (n = 81 in the testis network and n = 74 in the ovary network) (Table 4). In addition, among the network-specific hub genes, we identified SERPING1, CC chemokine receptor-5 (CCR5), TMPRSS2, and ADAM10 as the high-degree nodes within the testis-associated network, and BST2, GATA1, ENPEP, TLR4, TLR7, IRF1, and IRF2 within the ovary-related network. Detailed information is provided in Tables S1 and S2 of supplemental materials.

**Table 4:** Connectivity degrees in both PPI networks.

OVARIES		TESTICLES	
Gene name	Degree	Gene	Degree
**RDX**	80	**RDX**	92
**IRF2**	74	**HMGB1**	87
**HMGB1**	72	**RAB5C**	81
**RAB5C**	69	**IRF2**	81
RAB2A	64	GTF2F2	79

## Discussion

In this study we explored the gender variation in the transcriptional landscape of SARS-CoV-2 infection to provide an immediate understanding of variable susceptibility and COVID-19 manifestations in men and women. In fact, typical gene expression connected with predisposed tissue can claryfy the cellular response to the SARS-CoV-2 infection. In particular, expression of human proteins that interact with SARS-CoV-2 in the testicles and ovarian tissue and the relative interactions were analyzed in order to evaluate their potential association with sexual difference in the likelihood of infection and severity of symptoms. The analysis displayed that some genes expressed in the testis network were not present in the ovarian network and vice versa. Within the testis network we found TMPRSS2, ADAM10, SERPING1, and CCR5, while within the ovary network we found BST2, GATA1, ENPEP, TLR4, TLR7, IRF1, and IRF2 (See Supplementary materials Fig. S1 and Tables S1 and S2).

**Figure 3:**
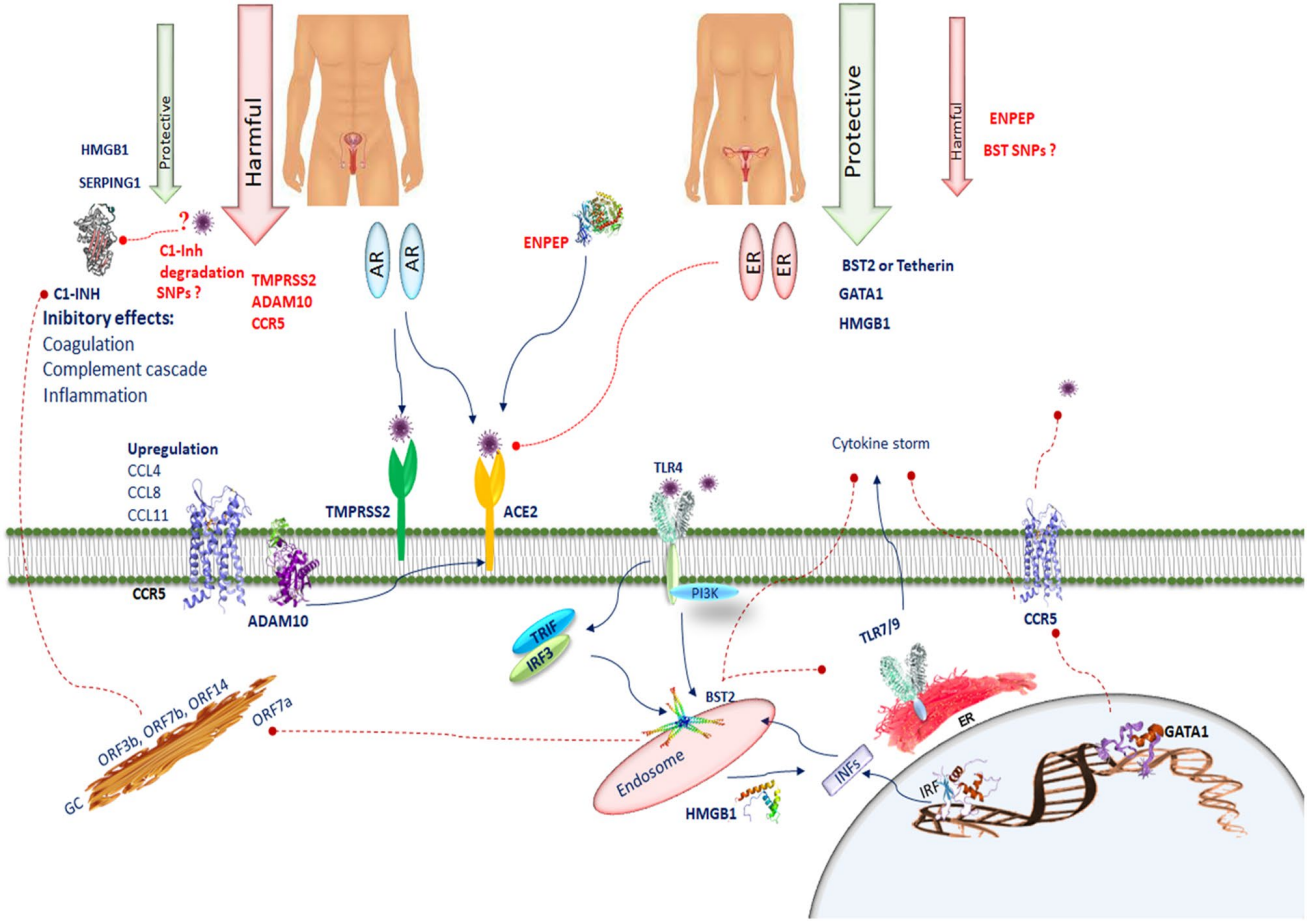
SERPING1 encodes C1INH, which suppresses complement and coagulation cascades and prevents inflammation. SERPING1 in the testis could prevent thrombotic risk. The interaction of several CoV2 proteins (ORF 3b, ORF7b, ORF14, nsp2ab, nsp13ab, nsp14ab and nsp8ab) with C1-INH can be inhibited during viral infection, leading to a predisposition to activate the complement cascade, the bradykinin pathway and the intrinsic coagulation cascade. The deletion in N-terminal region of SERPING1 by SARS-CoV-2 may block its function and increase inflammatory processes. Deteriorated SERPING1 expression caused by CoV2 interacting proteins could activate the intrinsic coagulation pathway, inducing a pro-coagulant state. CCR5 is involved in the pathology of SARS-CoV-2. In SARS-CoV-2 the chemotactic factors such as CCL4, CCL8, and CCL11 sharing CCR5 as a receptor are upregulated. ADAM10 is correlated with ACE2 cleavage regulation in human airway epithelia. BST2 reduces SARS-CoV-2 RNA replication. BST-2 is strongly induced after exposure to IFN through IRF1. BST2 expression is modulated by the TLR4/PI3K signaling pathway. Activation of TLR4 results in TRIF/IRF3-mediated positive regulation of BST-2. TLR7, TLR8 and TLR9 are predominantly localized in intracellular compartments and form the key gatekeepers in detecting and combating viral infections. In COVID-19 patients, host tetherin-mediated virion endocytosis may control TLR9 recognition to restrain immune cell responses. Several host factors such as the HMGB1 facilitate entry of self-DNA into the endosomes of pDCs, where they trigger TLR9 to induce type 1 IFN responses. GATA-1 is a potent repressor of CCR5 expression. CCR5 inhibition decreases IL-6 and SARS-CoV-2 plasma viremia.

Testicular tissue shows high ACE2 mRNA and protein expression levels^9^, which act as functional receptors for the coronavirus. In fact, to infect cells the COVID-19 spike adheres to ACE2 cell surface receptor^30^. Male sex hormones facilitate SARS-CoV-2 access into host cells affecting the ACE-2 pathway^31^. This priming is also carried out by TMPRSS2^13^. Activation of the androgen receptor increases TMPRSS2 levels through the androgen response element present in its promoter in many tissues. TMPRSS2 expression is greater in male lungs than in female lungs. In our analysis we found that TMPRSS2 was present in the testis. This may be one of the effects that could clarify the prevalence and severity of COVID-19 observed in men^32^. ACE-2, like TMPRSS2, is regulated by the androgen receptor. Therefore, it is possible that lowering androgen hormones could reduce the transcription of TMPRSS2, probably by decreasing the expression of ACE-2^33^. However, estrogen can contribute to the protection against virus infection, as demonstrated by a previous study exploring the role of sex hormones in the survival rate of SARS‐CoV infected male and female mice^34^. In this investigation it was observed that ovariectomized female mice had a severe form of the disease compared to controls. Moreover, fulvestrant, an estrogen nuclear receptor antagonist, reduced the survival rate in females. Instead, castrated male mice did not display an increased mortality rate due to SARS‐CoV infection compared to the control group, demonstrating that the predisposition to SARS‐CoV severity could be sex‐related, and estrogen may exet significant role in disease onset^35^. Therefore, estrogen can play a protective effect against COVID-19. Pre‐treatment with estrogens has a protective action in acute lung injury, as a result of the preventive anti‐inflammatory effects^36^. 17β‐estradiol modulates ACE2 gene expression levels, further supporting the role of sex hormones in COVID‐19^37^. Pulmonary NHBE cell line treated with 17β‐estradiol promoted a reduction in ACE2 gene expression. A link between the decrease in viral load and TMPRSS2 expression by estrogen treatment was also demonstrated. Despite the fact that no direct effects of estrogens on SARS‐CoV‐2 before cell infection were demonstrated, these hormones are able to decrease SARS‐CoV‐2 infection in vitro^38^. Angiotensin 1–7 may prevent ischemic cardiac damage and acute respiratory distress syndrome. In an animal experiment, long-term angiotensin 1–7 infusion resulted in strong antioxidant and vasodilating effects in female rats. But, the positive effect of long-term angiotensin 1–7 infusion in male rats was lacking^39^. It is likely that estrogen may strengthen the vasodilator and antioxidant properties of angiotensin 1–7. In addition, estrogen enhancement induces ER-α upregulation in T lymphocytes increasing the release of interferon I and III from T lymphocytes which alleviates the COVID-19 infection^40^ (Fig. 3).

### ADAM10, SERPING1 and CCR5 in the testis network

Disintegrin and Metalloproteases (ADAMs) belong to metzincin family of metalloproteases^41^. ADAMs are proteins ubiquitously expressed and regulate sperm–egg interactions, migration, cell proliferation, and differentiation. ADAM10 is expressed in the brain^42^ and control Central Nervous System (CNS) processes, such as development, synaptogenesis and axon targeting. At the synapse and in synaptic vesicles it acts as a sheddase of other synaptic proteins^43^ and regulates axon guidance and synaptic functions by leading the cleavage of synaptic proteins, such as Amyloid Precursor Protein, Neuronal Cell Adhesion Molecule and neuroligins. ADAM10 promotes microglia-mediated synapse removal by cleaving the chemokine fractalkine (CX3CL1), a ligand of CX3C chemokine receptor 1 (CX3CR1)^44^. In addition, ADAM10 is broadly expressed in intestinal epithelial cells^45^ and modulates intestinal permeability by repressing the transmembrane Notch proteins^46^ and E-cadherin which is one of the most important junction molecules involved in the preservation of structural integrity of the intestinal epithelial^47^. The Notch receptor is a substrate of ADAM10 and controls intestinal homeostasis^48^. Hence, the ADAM10-mediated shedding of the Notch receptor and E-cadherin downregulates epithelial cell migration and adhesion and protects intestinal barrier. In addition, ADAM10 is connected with ACE2 cleavage regulation in human airway epithelia^49^. Since ADAM10 regulates proteolytic cleavage of several key proteins acting in synapse formation, axon signaling and cell adhesion and regulating intestinal permeability it could aid in activating key pathways that are altered in COVID-19 pathogenesis.

Our analyses show a selective involvement of SERPING1 belonging to the superfamily of serine proteinase inhibitors, which encodes protein C1 inhibitor (C1INH), in the testis-associated network. SERPING1 was selected as one of the gene targets for analysis in this study since C1INH plays a critical role in stopping the activity of the first component of the complement (C1). Inhibition of C1 prevents the activation of complement components 2 and 4 (C2 and C4) as well as a number of downstream effects on the complement cascade^50^. Once SARS-CoV-2 infects human host, the complement system is promptly activated in order to restrain the infection. However, as observed for other pathogens, complement activation can be eluded and the infection takes over. The role of the complement system in the SARS-CoV-2 infection can be a double-edged sword. COVID-19 recurrently exhibits a hyper-coagulable inflammatory condition, presenting high levels of inflammatory cytokines, D-dimers^51^, fibrinogen^52^ and mild thrombocytopenia^53^. Post-mortem pathology studies report a high incidence of venous thromboembolism^54^ and micro vascular thrombi^55^ in the lungs and kidneys with endothelial swelling, consistent with a thrombotic microangiopathy. In parallel, robust complement activation has been observed in endothelial cells^56^. The complement and coagulation systems exhibit cross-talk, moreover, some evidence describes a link between the activation of the complement system and thrombosis in COVID-19 patients. In the course of the Sars-CoV2 infection, the complement system can be activated via multiple pathways by the virus itself and by damaged tissues. The generation of C5a, whose levels are highly elevated in symptomatic COVID-19 patients (in combination with a strong expression of C5aR in monocytes and neutrophils), increases tissue factor activity both in circulation^57^ and on endothelial cells^58^. The induction of endothelial P-selectin by C5a^59^ is essential for the enrollment and aggregation of platelets. C5a induces a large production of neutrophil extracellular traps (NET)^60^, which capture platelets causing platelet aggregation, coagulation and thrombus formation. Patients with severe COVID-19 display elevated serum markers of neutrophil activation and NET formation^61^. Endothelial cells are stimulated by MAC to produce von Willebrand factor^62^, which increases prothrombinase activity resulting in fibrin deposition and endothelial injury. It has been shown that MASP-1 and MASP-2 cleave prothrombin^58^ and activate fibrinogen and factor XIII^63^. A strong deposition of C4d, MASP-2 and MAC has been found in the lung and in the dermal microvasculature from COVID-19 patients with acute respiratory distress syndrome (ARDS)^64^. The interaction between the complement and coagulation systems could promote a inflammatory thrombotic state in COVID-19 patients. Although complement activation is decisive to control infection in asymptomatic or mild cases, intensified activation can produce exacerbations of the disease promoting cytokine storm, tissue damage, thromboembolism and other clinical manifestations of pathological coagulation such as disseminated intravascular coagulation (DIC) seen in patients with severe COVID-19^65^. C1INH acts in the control of the kinin-bradykinin system, coagulation and thrombolysis. It prevents several other serine proteinases including plasmin, kallikrein, and coagulation factors XIa and XIIa^66^. Overall, these evidence clearly indicate that the expression of SERPING1 in the testis should have a protective role for thrombotic risk. However, the epidemiological data on gender differential outcomes are against this notion. To explain the increased propensity of thrombosis in men it can be assumed that SARS-CoV2 proteases may have the capability to cleave the N-terminal region of SERPING1. The removal of carbohydrate moieties has perhaps no consequences on the SERPING1 role on kallikrein inhibition^67^, however, it may inhibit the complex formation with C1s/C1r proteases and the successive clearance of SERPING1-C1s/C1r complexes by rLDL related protein. The deletion of these sites from the SERPING1 molecule may neutralize its function and increase the inflammatory process. Another suggestion that might support the hypothesis on the possible interaction between the proteases of COVID19 and SERPING1 is that patients with SARS-Cov2 could express high cleaved C1INH, such as that was found in the serum of a patient with HIV-1^68^. Indeed, it has been reported that C1-INH is an interactor of 7 different CoV1 proteins and polypeptides, encoded by ORF3b, ORF7b, ORF14, nsp2ab, nsp13ab, nsp14ab and nsp8ab. These CoV1 proteins are comparable to their homologous CoV2 proteins^69^. C1INH degradation by plasmin may constitute a serious event in the defeat of protease inhibition during inflammation^70^ through lowering fibrinolysis and increasing thrombus formation. C1-INH is one of the proteins with the highest connectivity in the merged CoV2 interactomes. The interaction of several CoV2 proteins with C1INH showed that it can be repressed during viral infection, leading to a predisposition to trigger the complement cascade, the bradykinin pathway and the intrinsic coagulation cascade^71^. An impaired SERPING1 expression caused by CoV2 interacting proteins could stimulate the intrinsic coagulation pathway, inducing a pro-coagulant state that can overcome physiological anticoagulant activities (Fig. 3).

In agreement with the observation that host cell resistances to viral infections are based on chemokine and cytokine signals, the testis-related network analysis showed the implication of CCR5. It is a receptor for several CC chemokines that regulate leukocyte migration and activation. In the immune system, CCR5 is mainly expressed on CD4 + effector and memory T cells, natural killer T (NKT) cells, Th17, and macrophages, immature dendritic cells (DCs) and in bone marrow precursor cells during hematopoiesis^72^. CCR5 may promote inflammation in a wide range of infectious diseases by recruiting leukocytes towards inflammation sites^73^. In detail, CCR5 is more greatly expressed on interferon-γ (IFNγ)–secreting type 1 (Th1) cells than interleukin 4 (IL-4)–producing type 2 (Th2) cells^74^. CCR5 is involved in the pathology of SARS-CoV-2^69^. SARS-CoV-2 has a capacity to enter to the cells rapidly. Therefore, in SARS-CoV-2 infection the function of the CD8 T cells that are cleaning the infected cells is very critical. From birth to old age higher numbers of CD4 + T cells and CD4/CD8 T cell ratios are present in females^24^ Pathologically overproduced chemokines and their own receptors have been found in patients with COVID-19. These different chemotactic receptor expression profiles observed on Th1 and Th2 cells seem to be critical for regulating their migratory inclinations to various sites of inflammation and effect cell-mediated immune responses crucial for the control of the COVID-19 infection. SARS-CoV-2 infected lungs displayed an upregulation of chemotactic factors, including CCL4, CCL8, and CCL11, which all shared CCR5 as their receptor. CCL4 exhibits chemoattractive capacity towards different cell types such as immune cells, and coronary endothelial cells^75^. CCL5 and its receptor CCR5 are significantly induced in the infarcted myocardium and are associated with a higher risk of stroke and cardiovascular events^75^. Moreover, CCR5 and CCL5 play important roles in respiratory infections and inflammatory response, which frequently requires the recruitment of immune cells such as activated NK, CD8^+^ T cells and macrophages^76^ to remove infectious agents. Differential expression of chemokine receptors and ligands may modulate inflammatory patterns, which in turn may influence the establishment or progression of infections. Nevertheless, an intense increment of plasmatic levels of IL-6 and CCL5 (also known as RANTES), a ligand for CCR5, decreased CD8 + T cell levels, and SARS-CoV-2 plasma viremia in severe COVID-19 patients^77^. An improved chemotaxis created by unregulated CCL5 and cytokines, such as IL-6 and TNF-α, induces an inflammatory cascade leading to ARDS and multisystem organ failure^76^.

### BST2, GATA1, ENPEP, TLR4, TLR7, IRF1, and IRF2 in the ovary network

In our analysis the bone marrow stromal antigen 2 (BST2; also known as CD317 or tetherin) was found in the ovary-related network. BST2 is a virus restriction factor that has been recognized to be a powerful inhibitor of SARS-CoV-2 replication^77^. BST2 is circumscribed at the plasma membrane and in endosomes and acts through the ER and Golgi complex. It prevents viral release of numerous enveloped viruses, including human coronavirus and SARS-CoV-1, which bud at the plasma membrane or the ER and Golgi complex by tethering their virions to the cell surface or intracellular membranes^78^. BST2 prevents viral egress and antagonize the protein accessory SARS-Cov-2 Orf7a for virion release, hence, is responsible for tethering nascent enveloped SARS-CoV-2 virions to infected cell surfaces^79^. The transmembrane tetherin protein is expressed in bone marrow stromal cells, B-cells, dendritic cells, and other cell types^79^ and is found predominantly in the cholesterol-rich domains (lipid rafts) of the plasma membrane^78^. BST-2 is robustly induced on the surface of many types of cells after exposure to IFN through the IRF1^80^ and other proinflammatory cytokines via STAT activation^81,82^. As a result, BST-2 prevents the release of formed viral particles inside the cell avoiding their spread. IRF1 controls constitutive antiviral gene networks to counterattack viral infections in human respiratory epithelial cells, by regulating early expression of IFNs^80^. Moreover, it stimulates constitutive expression of the anti-viral genes such as BST2*,* OAS2*,* and RNASEL, to preserve an excellent anti-viral state. Specifically, BST2 expression is modulated by the TLR4/PI3K signaling pathway. Activation of TLR4 generates the TRIF/IRF3-mediated positive regulation of BST-2 whereas MYD88/PI3K exerts a negative regulation^83^. Among the TLR family, TLR3, TLR7, TLR8 and TLR9 are principally localized in intracellular compartments and are specialist in identifying and counteracting viral infections^84^. TLR9 identifies RNA and DNA motifs enriched in unmethylated Cytosine-phosphate-Guanine (CpG) sequences, which are expressed in the bacterial and viral genome^85^. Human mitochondrial DNA (mtDNA), evolutionarily derived from endosymbiont bacteria, contains unmethylated CpG-motifs and is a model of a well-known DAMP that initiates inflammatory process directly via TLR9 in the course of injury and infection^86^. mtDNA, formed from damaged host cells, also modifies self-ligands, called carboxy-alkyl-pyrrole protein adducts (CAPs), which are formed during oxidative stress, contribute to intensify TLR9/MyD88 pathway activation infection^86^. CAPs promote platelet activation, granule secretion, and aggregation in vitro and thrombosis in vivo^87^. Since circulating mtDNA levels rise with old age they are a familiar feature contributing to chronic inflammation in elderly (termed “inflamm-aging”)^88^. In the context of COVID-19, this TLR9 axis of inflamm-aging could have consequence, just because older age is associated with high risk of developing severe complications of COVID-19. In the course of COVID-19, TLRs are essential in the viral battle. Specifically, as regards the role of TLR9 in resistance against SARS-CoV-2 it has been proposed that there is an excessive TLR9 activation in severe COVID-19 pathology. TLR9 is largely expressed in different cell types including epithelial cells in the lungs and nasal mucosa, in muscles and brain, on plasmacytoid dendritic cells and B cells, monocytes, macrophages, neutrophils, T lymphocytes, NK cells, megakaryocytes and platelets^89^. Regarding TLR9 COVID-19 theory suggests that in particular susceptible patients, the activation of TLR9 could be a soft but powerful force inducing hyperinflammation and thrombotic complications seen in SARS-CoV-2. Hence, the efficacy of TLR9 agonists in defense against SARS-CoV-2 is advisable^90^. BST2 expression is regulated by inflammatory signals and its stimulatory receptors may control immune functions by interacting with inducible or pathogen-associated ligands. It has been proposed that endocytosis-competent Tetherin may induce the intracellular uptake of virions into endosomes blocking viral release^91^. TLR9 is located in endosomes. Through the TLR9/MyD88 pathway, can be activated in various cell types such as Th1 and Th17 lymphocytes, B cells, dendritic cells, neutrophils and platelets inducing various inflammatory mediators such as type 1 IFNs, TNFa, IL-6, IL-8, IL-10, and IL-17^87^ (Fig. 3). All these cell types and intermediaries promotes the cytokine storm and thrombotic complications observed in the multi-organ failure in patients with severe COVID19 infections^92^. It has been proved that BST2 promotes NK cell, CD4 + T cell and CD8 + T cell response^93^. Moreover, BST2 modulate IFN responses of human pDC through ILT7^94^. These vigorous cell-mediated immune responses associated with lower infection levels indicate that tethering-mediated retrovirus control activities by modulating adaptive immunity. Tetherin acts on different phases of cell-mediated immunity, but depend on antigen presenting cells for activation. Tetherin + DCs are more vigorously stimulated virus-specific CD4 + T cells compared to Tetherin KO DCs ex vivo even though similar virus infection levels. Tethering-mediated virion aggregation on the cell surface can be a mechanism required to increase NK cell-mediated killing^95^. Internalization of tethered virions could stimulate viral sensing by endosomal sensors and successive upregulation of cytokines essential for NK cell function such as IL15. Thus, it is coinceivable that specific health conditions of the host tetherin-mediated virion endocytosis may control TLR9 recognition to restrain immune cell responses. This may induce production of cytokines such as type I IFN and IL15, which can both trigger NK cell responses. In mice, Tetherin enhances type I IFN expression in plasmacytoid dendritic cells (pDC), after stimulation with virus in vitro^96^. The pDC repertoire of PRRs is very specialized and includes mainly TLR7 and TLR9. Endosomes of pDCs trigger TLR9-mediated induction of type 1 IFN responses^97^. As a result of their higher type I IFN production and nucleic acid-oriented sensing of pathogens through endosomal TLRs, pDCs exhibit vast antiviral functions and are slightly tolerant to viral infections compared to cDCs^98^. Since that BST2 serves as a physiological ligand for the human pDC-specific receptor ILT7 it may act as a critical link between type I interferons and pDC responses in retrovirus infections. Human pDCs express both inhibitory receptors and stimulatory receptors^99^. pDC receptors ILT7, BDCA2, high-affinity Fc receptor for IgE, and NKp44 have negative effects on the IFN response of pDC to TLR activation through the ITAM-mediated pathway^94^. Intracellular TLRs have a partial aptitude to identify host versus foreign nucleic acids^100^. Numerous host factors, including antimicrobial peptide LL37, anti-DNA antibodies or the nuclear DNA-binding protein HMGB1, alone or in combination, promote entry of self-DNA into the endosomes of pDCs, where they activate TLR9 to induce type 1 IFN responses^97^. In the same way, autoantibody small nuclear ribonucleoprotein complexes can trigger TLR7 via FcγRII to induce IFN^101^. BST2 is the first non–MHC class I–type ligand for a member of the ILT receptor family^102^. pDCs are involved in antiviral innate immune responses by secreting large quantities of IFN-α/β. Nevertheless, type 1 IFN responses immediately after viral infection are short lived. Since BST2 is induced on the surface of many types of cells after exposure to IFN and via STAT activation^81^, BST2–ILT7 interaction may act as an indispensable negative feedback mechanism for avoiding sustained IFN synthesis after viral infection. Experimental evidence demonstrated that BST2 expression significatively decreased SARS-CoV-2 RNA replication (53% less than control cells) followed by a more effective decline (74%) of viral release^92^. Additional studies should further explain the role of BST2 in affecting viral pathogenesis and host cell-mediated immune responses.

At first GATA1 was recognized as a trans-acting factor of globin and other erythroid-specific genes. GATA-1 is expressed in CD34 + hematopoietic stem cells (HSCs), erythroblasts, megakaryocytes, and mast cells, basophils and eosinophils, regulating megakaryopoiesis and erythropoiesis^103^. It is well known the solid link between the GATA-binding protein members and T-cell differentiation. GATA-1 trans-activates CCR5 promoter activity in a transformed T-lymphoid cell line^98^. CCR5 includes DNA binding sites for GATA TFs^104^. GATA-1 decreases CCR5 promoter activity and hence is a powerful repressor of CCR5 expression at both protein and transcript levels in human T-cell subsets and DCs^104^. The suppression of CCR5 in human T cells by GATA-1 further confirm the idea that GATA-1 acts as an efficent and selective transcriptional repressor. Ectopic expression of GATA-1 prevents the expression of CCR5 and other Th1 effector molecules such as IFN-γ and CXCR3 and anti-inflammatory cytokines IL-4, IL-5, IL-13 and chemotactic receptors such as CCR4 and CRTH2^105^. The non-conserved trans-activation domains of GATA-1 have the function of supplementary protein interaction interfaces^106^, which could let it to selectively engage repressive cofactors to the IFNγ and CXCR3 promoters. The observation that GATA-1-responsive loci are epigenetically changed during hematopoiesis indicates that, in turn, GATA-1 may epigenetically modify the CCR5 promoter. The result that GATA-1 inhibits effectively CCR5 expression in numerous human cell types ^107^, strongly suggests that GATA-1 exerts the same repressor function in SARS-COV-2 target cells (Fig. 3). Therefore, GATA-1, which is expressed in HSCs and is silenced during their differentiation to CCR5-expressing DCs^107^, could influence COVID-19 susceptibility of these cell types during hematopoiesis, as well as mast-cell progenitors, which are all potential targets of viral infection in vivo^108^. A deeper understanding of the mechanism by which GATA-1 represses CCR5 expression can help unravel the genetic regulation of CCR5 in the target cells of COVID-19 and may be valuable in devising new approaches to antagonize CCR5 in individuals with COVID-19 infection. These observations increase the opportunities to understand the mechanisms by which the GATA-1 intervenes in the suppression of CCR5 in order to identify the therapeutic strategy to make the host cells resistant to the infection of SARS-COV2. Blocking CCR5 should promote rapid decline of IL-6, re-establishment of the CD4/CD8 ratio, and a significant reduction in SARS-CoV-2 plasma viremia.

## Conclusion

To date, various data have shown that SARS-CoV-2 needs ACE2 and TMPRSS2 expression for viral activity and that androgens increase ACE2 and TMPRSS2 gene expression, whereas, estrogens reduce ACE2 mRNA expression. Therefore, viral entry seems to be facilitated in man. Nevertheless, the immunomodulatory effects of sex hormones are insufficient to explain the gender difference in susceptibility, severity and mortality to COVID-19.

Our bioinformatic findings about the SARS-CoV-2 effects on gonads demonstrate, for the first time, that in female gonads the gene associated with the host immune response against COVID-19 viral infections are prevalent compared to those found in the male gonads (see supplemental material). In particular, the expression of BST-2 and GATA-1 and their networks strongly suggest that women have stronger cell-dependent and humoral responses to infection, resulting in a more rapid pathogen elimination in females than in males.

On the other hand, the expression of Serping1, observed in male gonads, could be a valuable protective factor against the onset of the cytokine storm. However, obviously, only this factor is not enough to counteract the adverse effects caused by the infection of COVID-19. Mainly genes that promote the susceptibility and strenghthen the severity of SARS-CoV-2 are expressed in male gonads (see supplemental material). Our studies encourage an urgent need to analyze the expression of these particular genes in promoting or suppressing viral load in SARSCoV-2 and the cytokine storm in infected experimental models. For example, it could be interesting to investigate the influence of genetic polymorphism relevant to identify the involvement of several genes especially Serping 1 or BST2 on the severity of COVID-19. Overall, this finding contributes to explain gender difference in susceptibility, severity and mortality to COVID-19 and provide potential diagnostic and therapeutic targets.

## Supplementary Information

Supplementary Information.

## Data Availability

Publicly available datasets were analyzed in this study. These data can be found here: https://www.proteinatlas.org/humanproteome/sars-cov-2.

## Abbreviations

ACE 2: Angiotensin-converting enzyme 2
ADAM10: Disintegrin and metalloproteinase domain-containing protein 10
ANPEP: Alanyl Aminopeptidase, Membrane
AR: Androgen receptors
BST2: Bone marrow stromal antigen 2
CCL: Chemokine
CCR5: Chemokine receptor-5
C1INH: C1 inhibitor
DPP4: Dipeptidyl peptidase-4
ENPEP: Glutamyl Aminopeptidase
ER: Estrogen receptors
FOXP3: Forkhead box P3
GATA-1: GATA Binding Protein 1
HMGB1: High Mobility Group1
IFN: Interferon
IL-6: Interleukin-6
IRF: Interferon Regulatory Factor
ORF: OPEN READING FRAM
TLR: Tolk like receptor
TMPRSS2: Transmembrane Serine Protease 2
