# Supplementary material for: Candidate genes of SARS-CoV-2 gender susceptibility
Source: Sci Rep. 2021 Nov 9;11:21968. doi: 10.1038/s41598-021-01131-7 (PMC8578384; doi:10.1038/s41598-021-01131-7)
Supplement: Supplementary file 1 — Supplementary Information. [file 41598_2021_1131_MOESM1_ESM.docx]

**Supplementary materials**

**Figure 1. Panel A: Interaction-specific views of gene networks of SARS-CoV-2 targeted human proteins expressed in testis and their interactions with the androgen receptor and the list of genes of our interest.** The figure shows different interaction-specific views of the PPI network constructed by GeneMANIA reporting the interconnection between SARS-CoV-2 targeted human proteins expressed in testis, the genes of interest and androgen receptor. The edges between nodes (proteins) indicate interactions based on the GeneMANIA database information. For each network, differently colored ‘edges’ indicate the type of evidence supporting each interaction: co-expression (light purple), physical interaction (pink), genetic interaction (green), shared protein domains (golden yellow), pathway (light blue), predicted (orange), and co-localization (blue). **Panel B: Interaction-specific views of gene networks of SARS-CoV-2 targeted human proteins expressed in ovary and their interactions with the estrogen receptors and the list of genes of our interest.** The figure shows different interaction-specific views of the PPI network constructed by GeneMANIA reporting the interconnection between SARS-CoV-2 targeted human proteins expressed in ovary, the genes of interest and estrogen receptors. The edges between nodes (proteins) indicate interactions based on the GeneMANIA database information. For each network, differently colored ‘edges’ indicate the type of evidence supporting each interaction: co-expression (light purple), physical interaction (pink), genetic interaction (green), shared protein domains (golden yellow), pathway (light blue), predicted (orange), and co-localization (blue).

**Testis Ovary**

**A B**

| **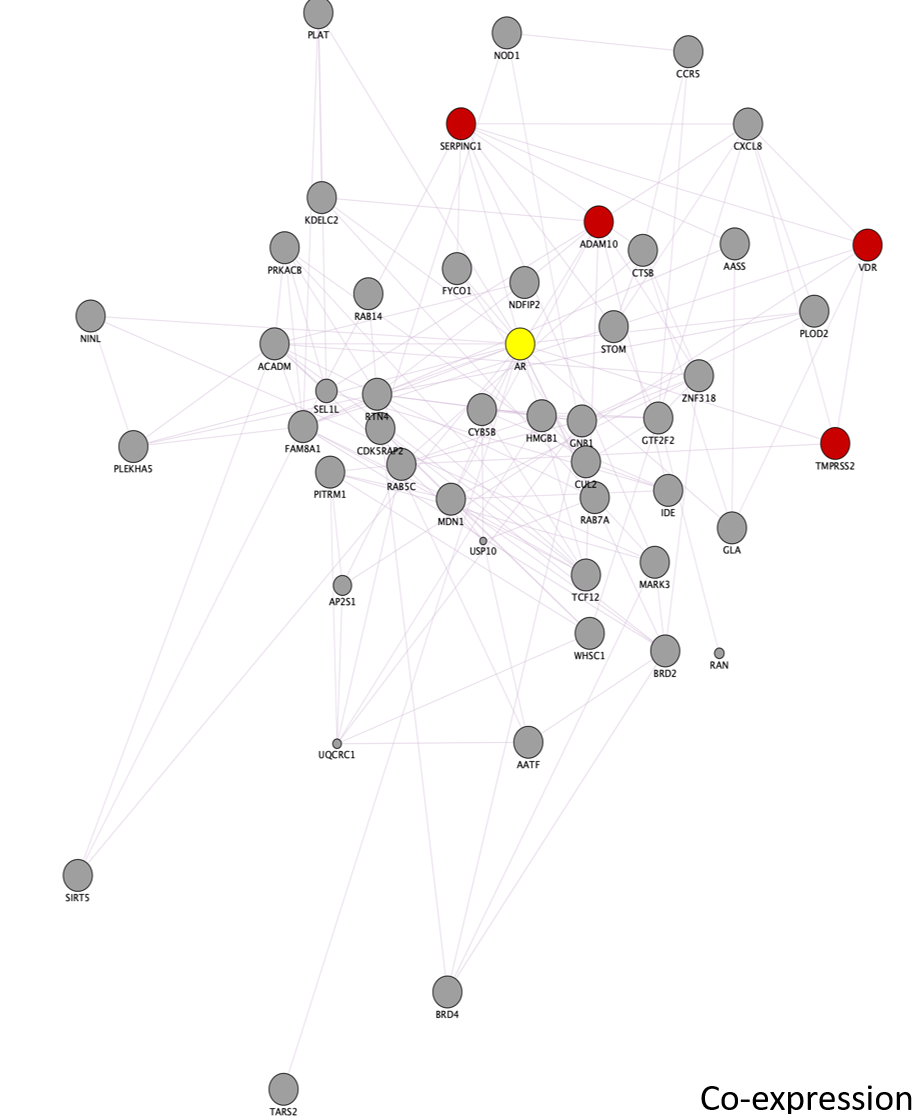** | **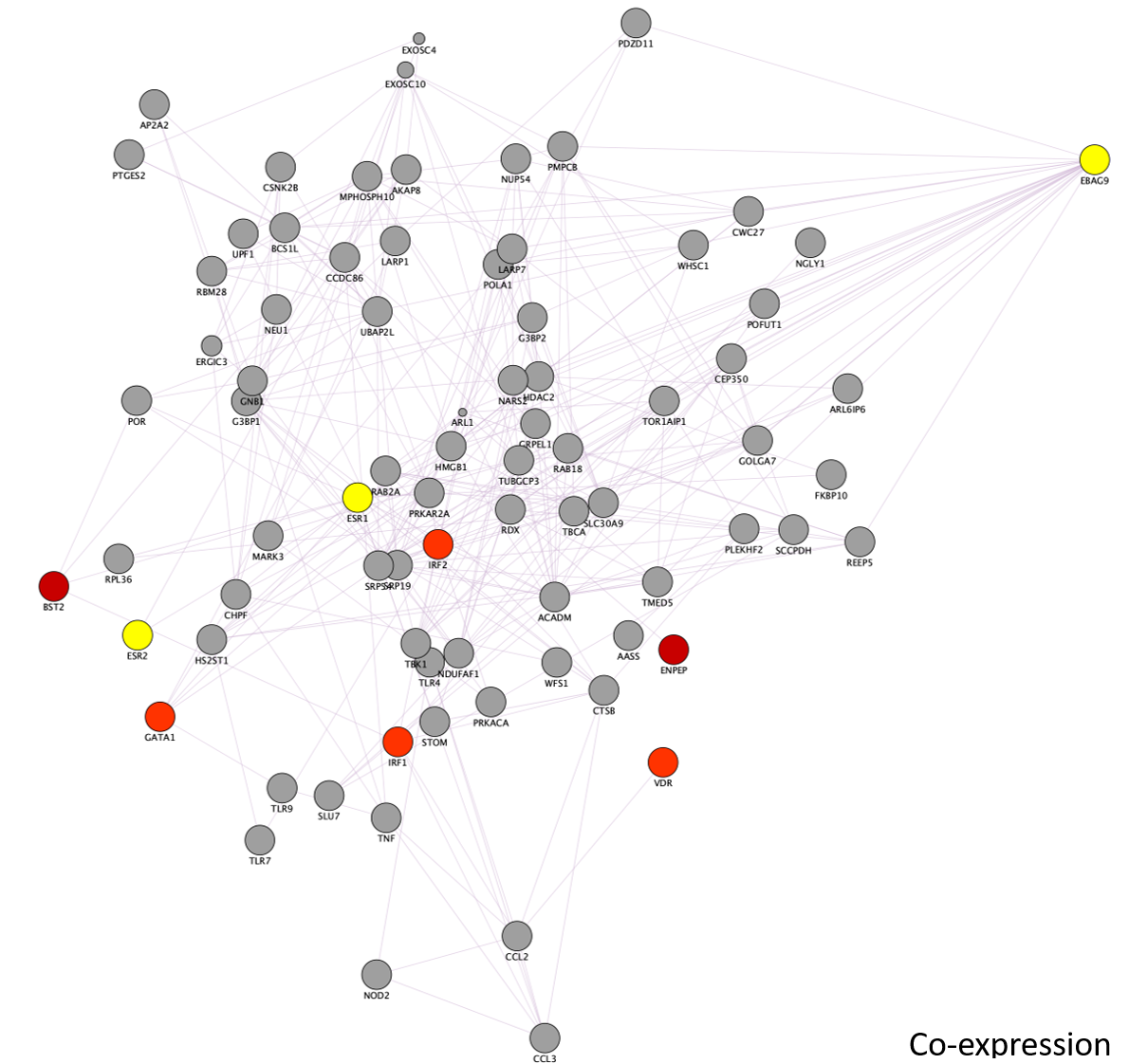** |
| --- | --- |
| **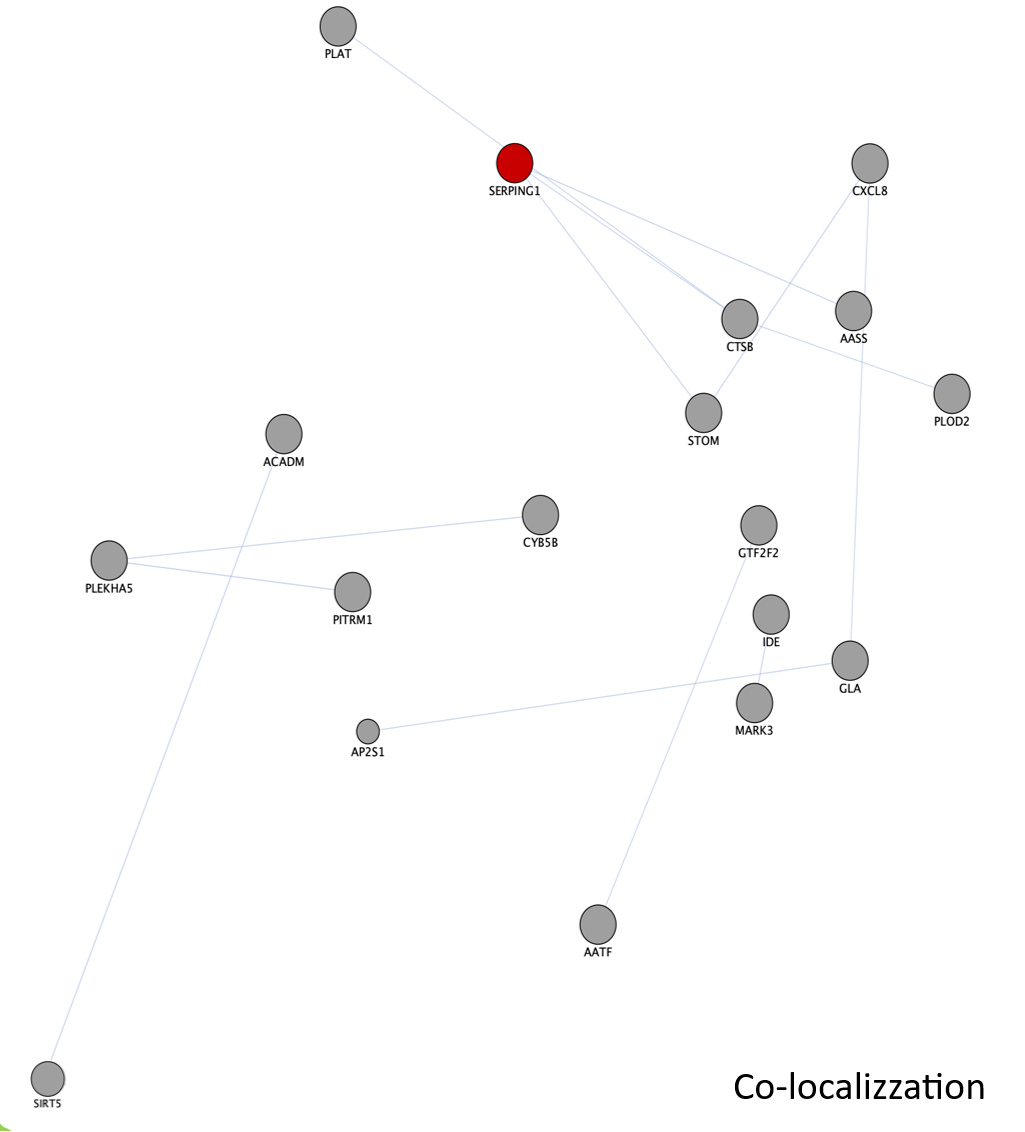** | **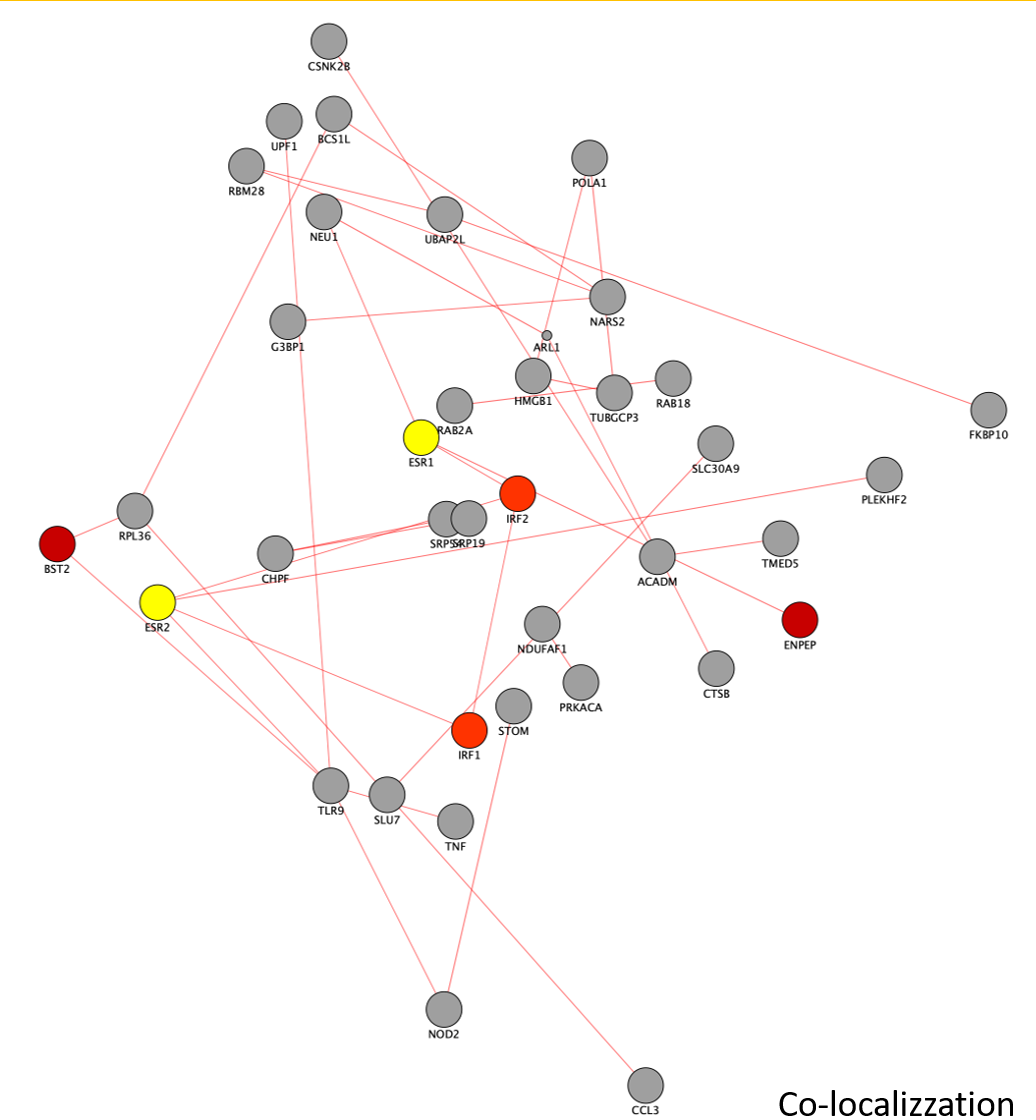** |
| **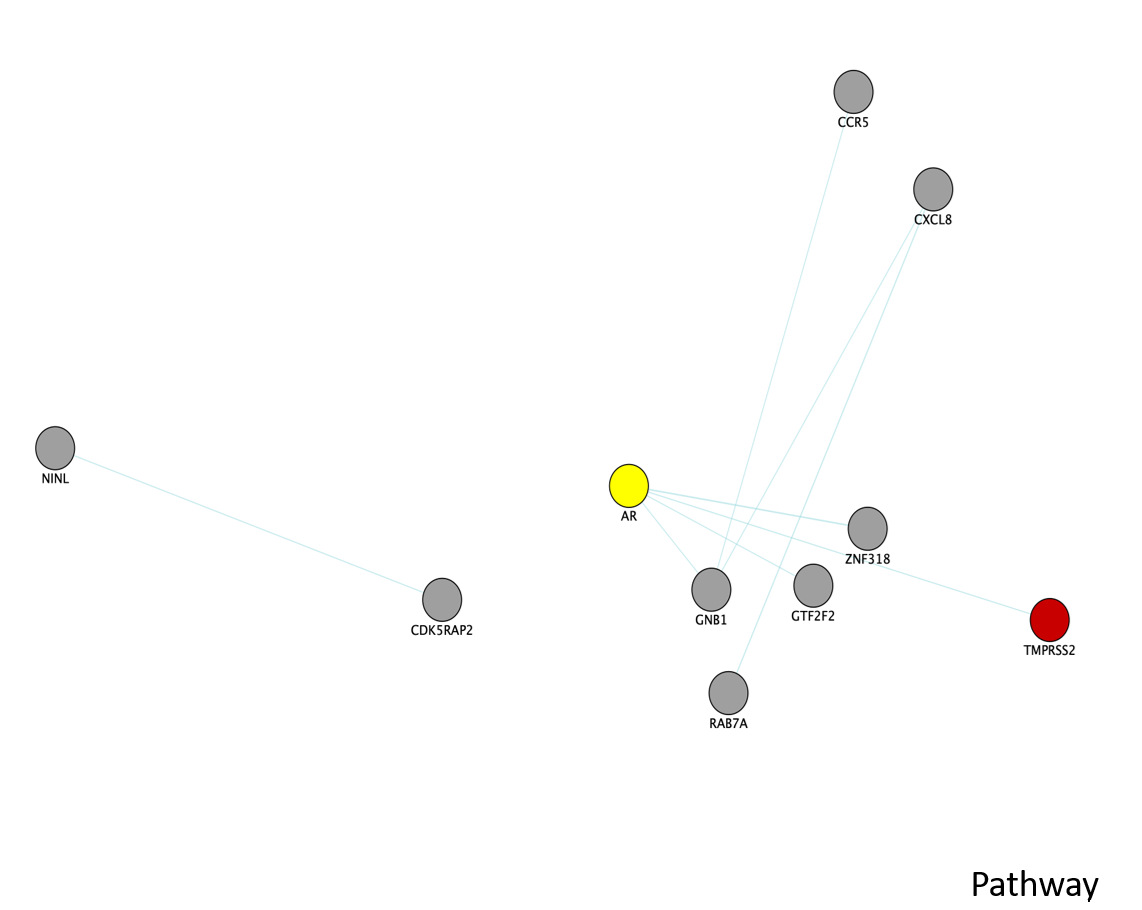** | **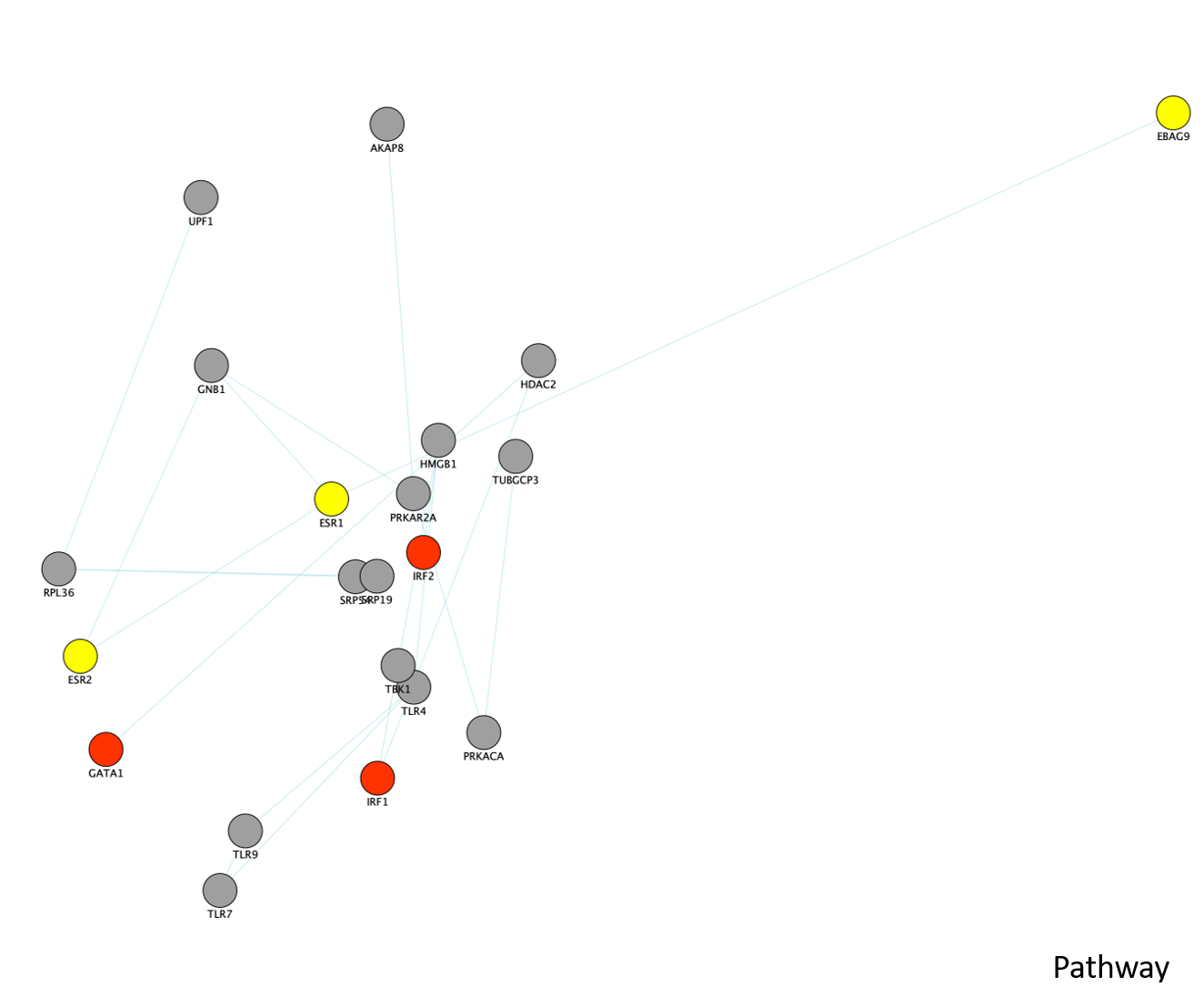** |
| **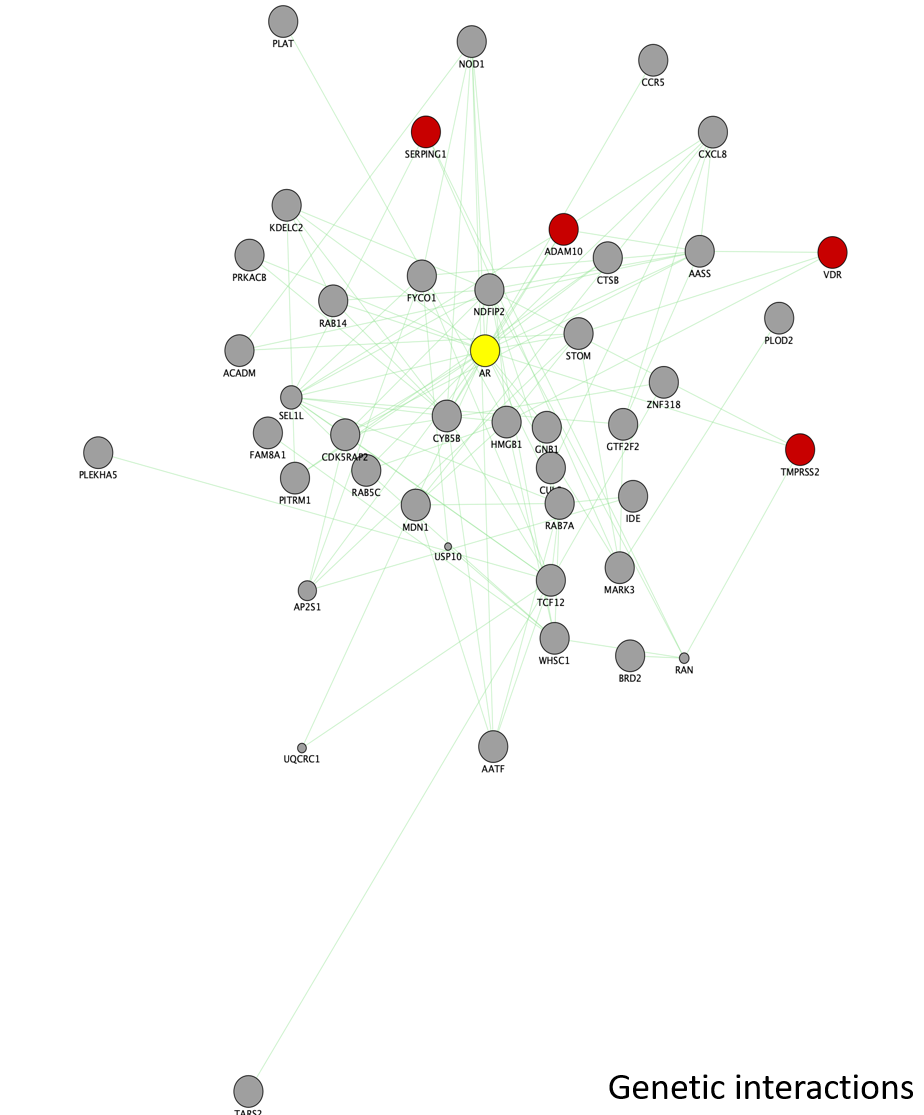** | **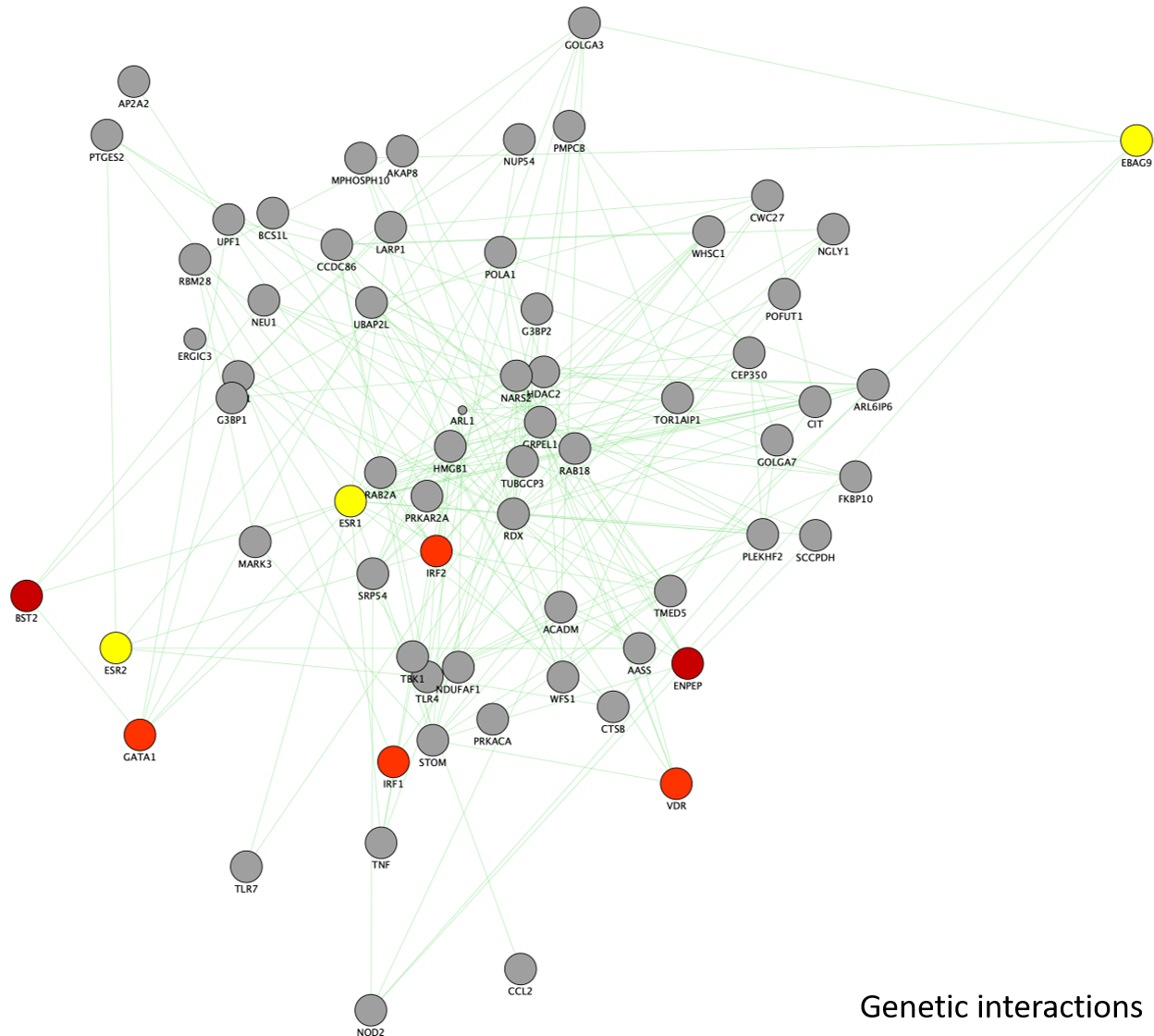** |
| **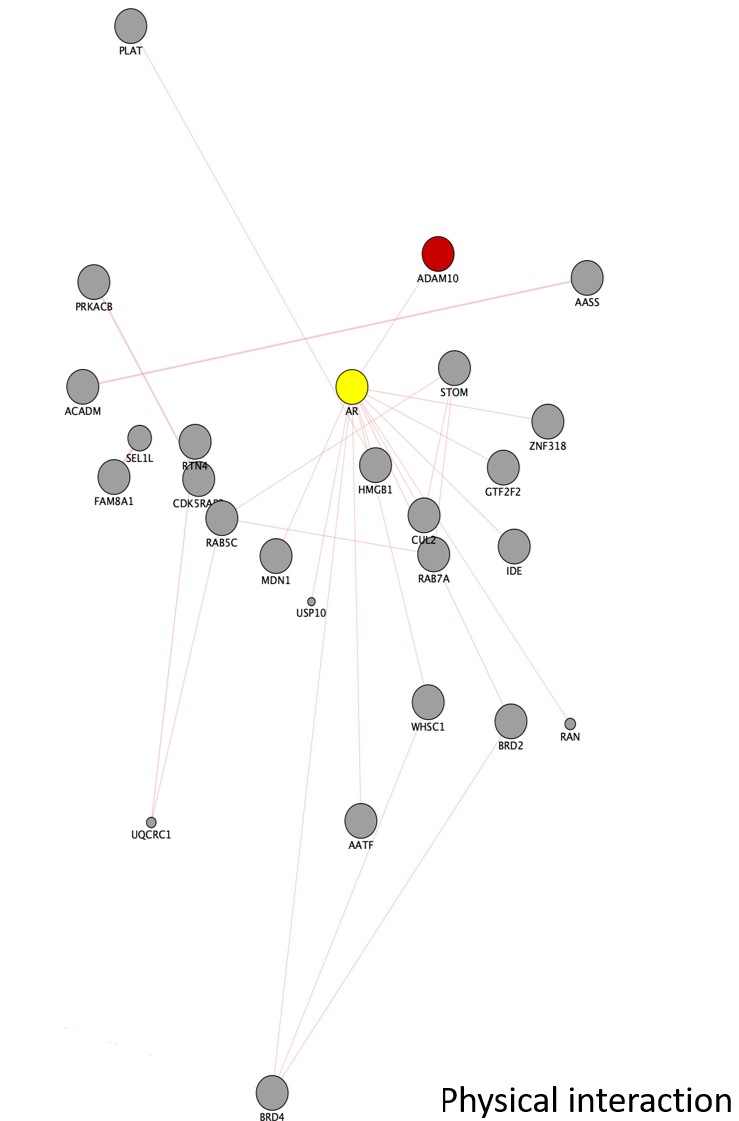** | **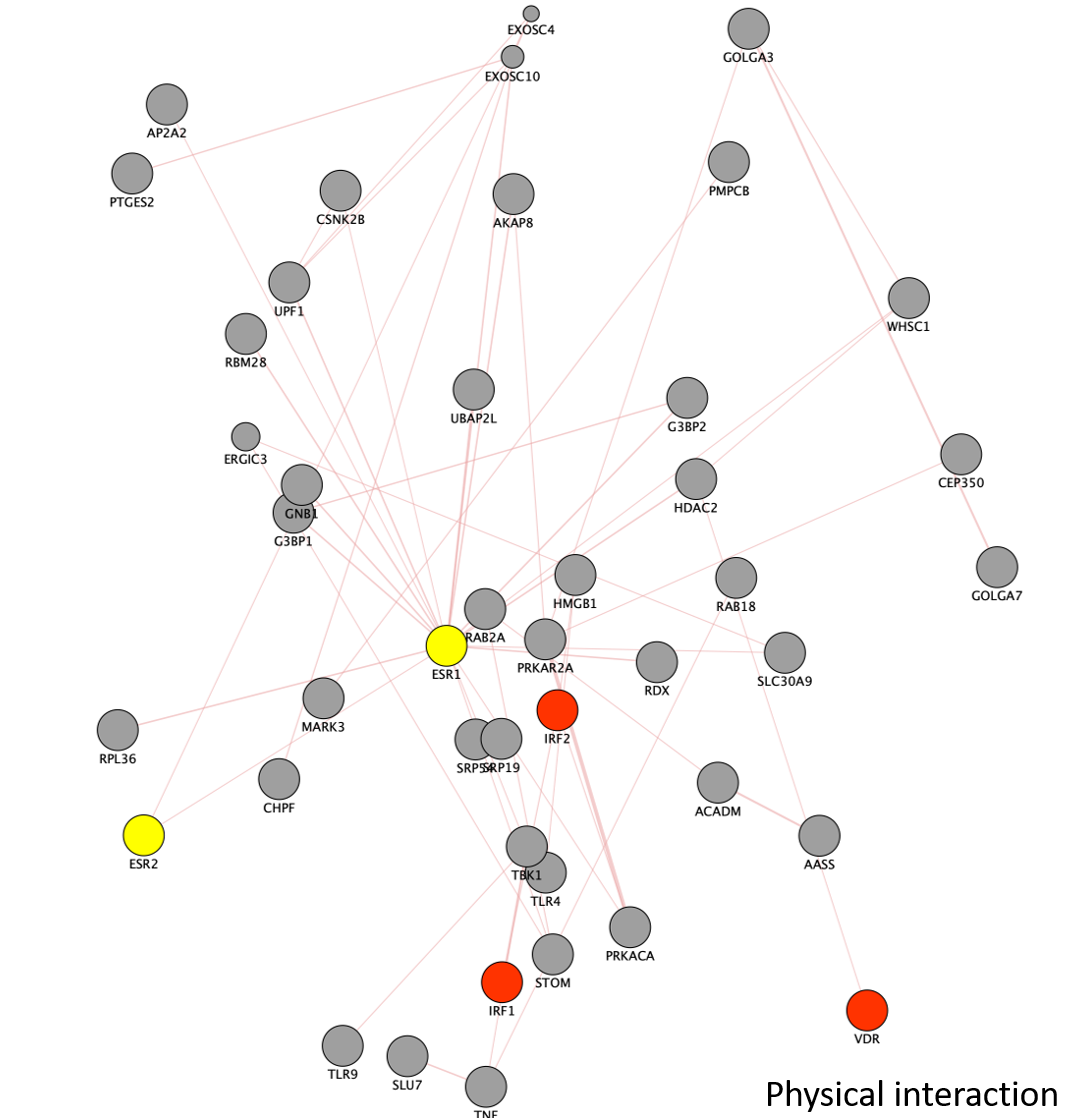** |
| **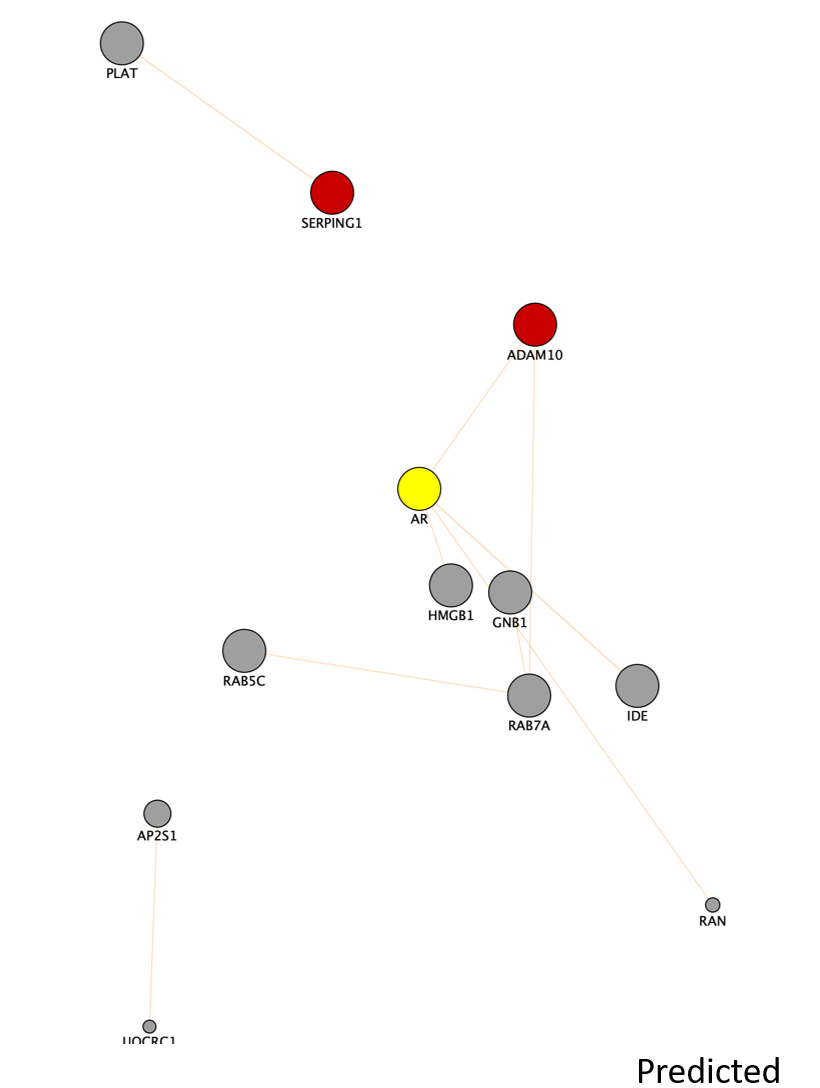** | **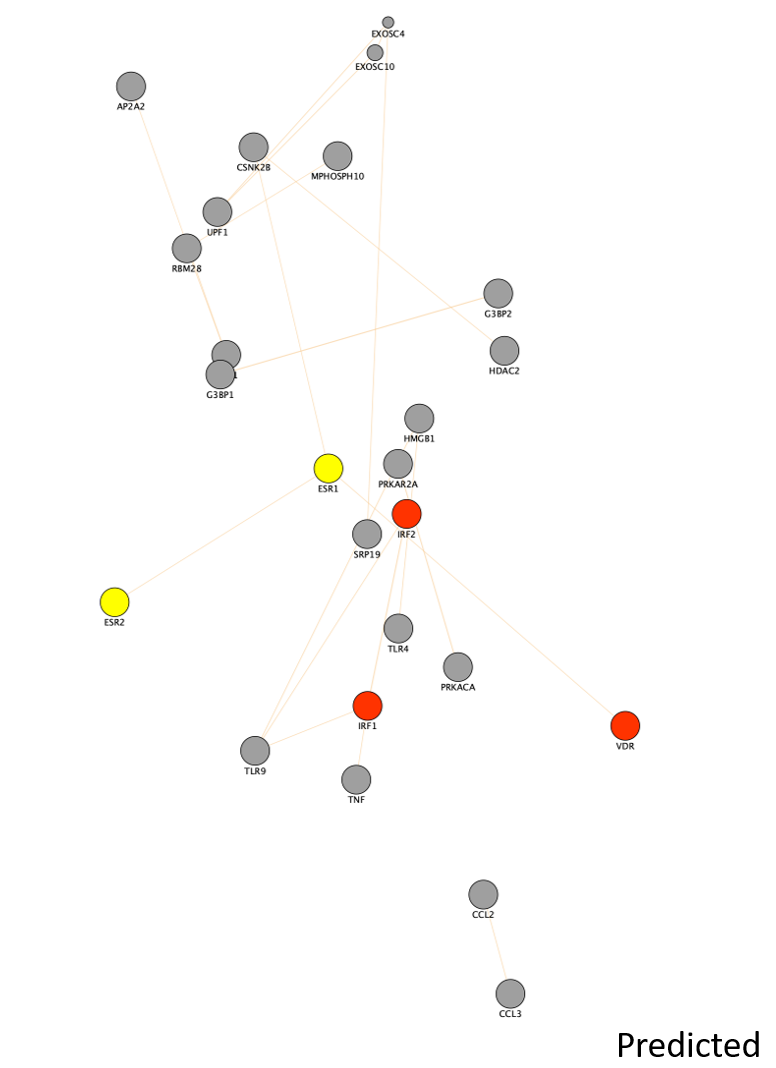** |
| **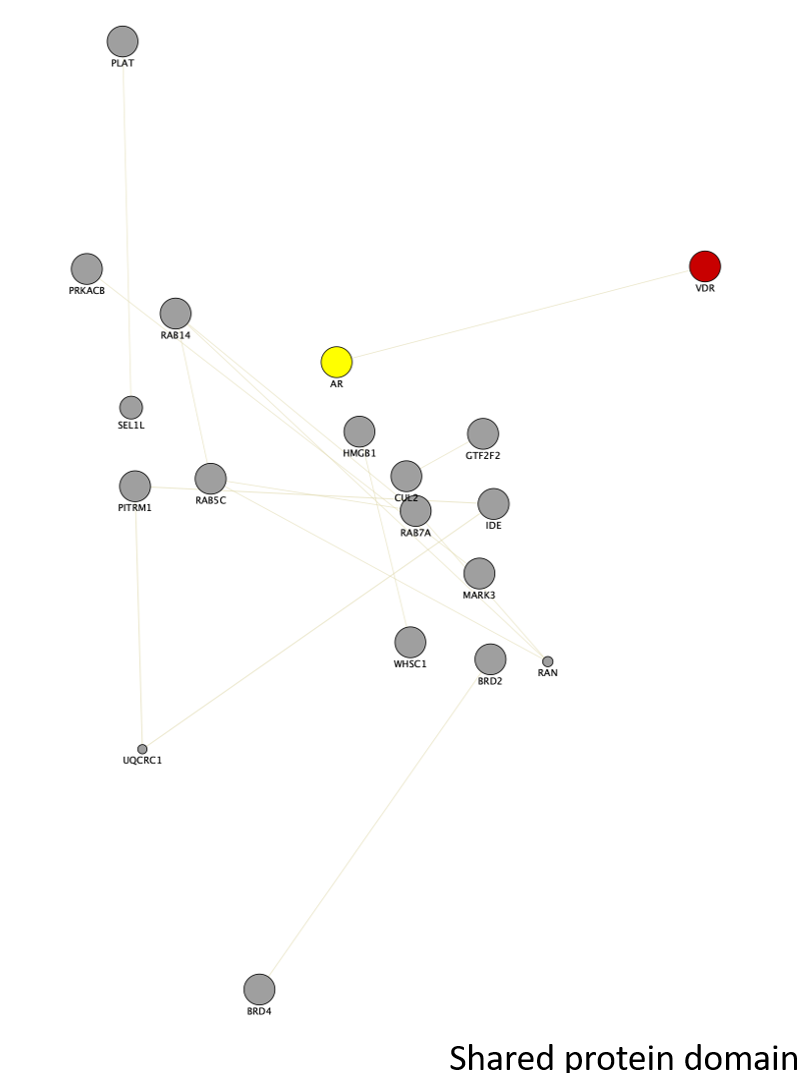** | **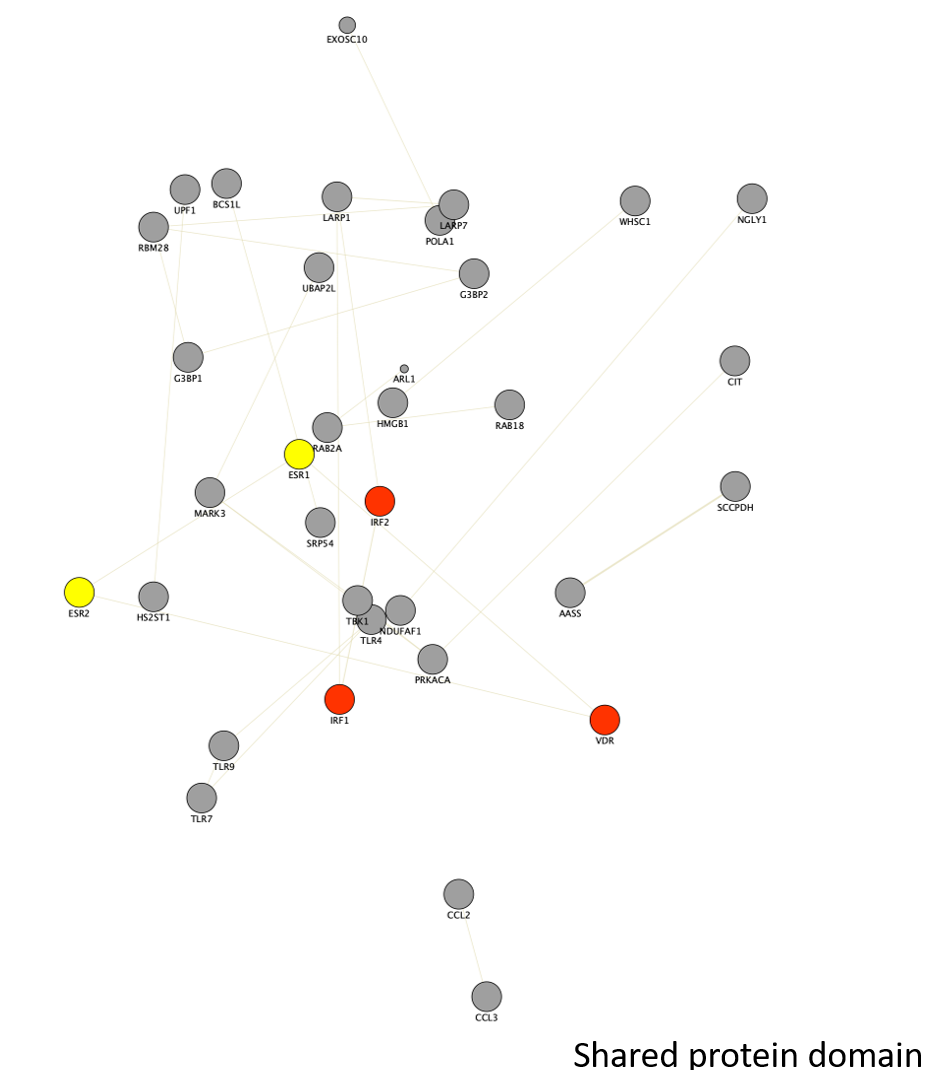** |

**Figure 1 S**

**Table S1.** Main genes in the testis network

| Testis network | Gene | Interaction |
| --- | --- | --- |
| SERPING1 | CTSB | co-expression  co-localization |
|  | CXCL8 | co-expression |
|  | AR | co-expression |
| CCR5 | NOD1 | co-espression |
|  | AR | genetic interaction |
|  | GTF2F2 | co-expression |
|  | GNB1 | pathway |
|  | CTSB | co-expression |
| TMPRSS2 | AR | co-expression  genetic interaction |
|  | RAB5C | co-expression |
|  | CXCL8 | co-expression |
|  | RAN | genetic interaction |
| ADAM 10 | AR | predict  genetic interaction |
|  | AASS | genetic interaction |
|  | RAB7A | predict |
|  | SEL1L | genetic interaction  co-expression |

**Table S2.** Main genes in the ovary network

| Ovary network | Gene | Interaction |
| --- | --- | --- |
| BST2 | RPL36 | co-localization |
|  | NEU1 | genetic interaction |
|  | POR | co-expression |
|  | TLR9 | co-localization |
|  | ESR1 | genetic interaction co-expression |
|  | IRF1 | co-expression |
| GATA1 | UBAP2L | co-expression  genetic interaction |
|  | HDAC2 | pathway |
|  | HMGB1 | genetic interaction |
|  | ESR1 | co-expression |
| ENPEP | RDX | co-expression |
|  | ESR1 | co-localization |
| TLR4 | NOD2 | co-expression |
|  | CCL2 | co-expression |
|  | CCL3 | co-expression |
|  | TLR9 | pathway  shared protein domain |
|  | TLR7 | pathway  shared protein domain |
|  | HMGB1 | pathway  predict |
|  | HS2ST1 | co-expression |
|  | TLR9 | pathway  shared protein domain |
|  | IRF2 | co-expression |
| IRF1 | IRF2 | predict  co-localization  co-expression |
|  | CTSB | co-expression |
|  | BST2 | co-expression |
|  | CCL2 | co-expression |
|  | CCL3 | co-expression |
|  | HDAC2 | pathway |
|  | TNF | predict |
|  | TLR9 | predict |
| IRF2 | HMGB1 | physical interaction pathway |
|  | ACADM | co-expression |
|  | ESR1 | co-localization |
|  | PRKACA | co-expression  physical interaction |
|  | SRP54 | co-expression |
|  | TBK1 | co-expression |
